# Changes in saliva protein profile throughout *Rhipicephalus microplus* blood feeding

**DOI:** 10.1186/s13071-024-06136-5

**Published:** 2024-01-27

**Authors:** Itabajara da Silva Vaz Junior, Stephen Lu, Antônio F. M. Pinto, Jolene K. Diedrich, John R. Yates, Albert Mulenga, Carlos Termignoni, José Marcos Ribeiro, Lucas Tirloni

**Affiliations:** 1 Centro de Biotecnologia, Universidade Federal do Rio Grande do Sul, Porto Alegre, RS Brazil; 2 Faculdade de Veterinária, Universidade Federal do Rio Grande do Sul, Porto Alegre, RS Brazil; 3 Vector Biology Section, Laboratory of Malaria and Vector Research, National Institute of Allergy and Infectious Diseases, Rockville, MD USA; 4 Clayton Foundation Peptide Biology Lab, Salk Institute for Biological Studies, La Jolla, CA USA; 5 Department of Molecular Medicine, The Scripps Research Institute, La Jolla, CA USA; 6 Mass Spectrometry Core, Salk Institute for Biological Studies, La Jolla, CA USA; 7 Department of Veterinary Pathobiology, College of Veterinary Medicine, Texas A&M University, College Station, TX USA; 8 Departamento de Bioquímica, Universidade Federal do Rio Grande do Sul, Porto Alegre, RS Brazil; 9 Tick-Pathogen Transmission Unit, Laboratory of Bacteriology, National Institute of Allergy and Infectious Diseases, Hamilton, MT USA

**Keywords:** Tick-host interaction, Parasite, Host proteins, Saliva, Sialoproteome

## Abstract

**Background:**

When feeding on a vertebrate host, ticks secrete saliva, which is a complex mixture of proteins, lipids, and other molecules. Tick saliva assists the vector in modulating host hemostasis, immunity, and tissue repair mechanisms. While helping the vector to feed, its saliva modifies the site where pathogens are inoculated and often facilitates the infection process. The objective of this study is to uncover the variation in protein composition of *Rhipicephalus microplus* saliva during blood feeding.

**Methods:**

Ticks were fed on calves, and adult females were collected, weighed, and divided in nine weight groups, representing the slow and rapid feeding phases of blood feeding. Tick saliva was collected, and mass spectrometry analyses were used to identify differentially secreted proteins. Bioinformatic tools were employed to predict the structural and functional features of the salivary proteins. Reciprocal best hit analyses were used to identify conserved families of salivary proteins secreted by other tick species.

**Results:**

Changes in the protein secretion profiles of *R. microplus* adult female saliva during the blood feeding were observed, characterizing the phenomenon known as “sialome switching.” This observation validates the idea that the switch in protein expression may serve as a mechanism for evading host responses against tick feeding. Cattle tick saliva is predominantly rich in heme-binding proteins, secreted conserved proteins, lipocalins, and protease inhibitors, many of which are conserved and present in the saliva of other tick species. Additionally, another remarkable observation was the identification of host-derived proteins as a component of tick saliva.

**Conclusions:**

Overall, this study brings new insights to understanding the dynamics of the proteomic profile of tick saliva, which is an important component of tick feeding biology. The results presented here, along with the disclosed sequences, contribute to our understanding of tick feeding biology and might aid in the identification of new targets for the development of novel anti-tick methods.

**Graphical Abstract:**

**Figure:**
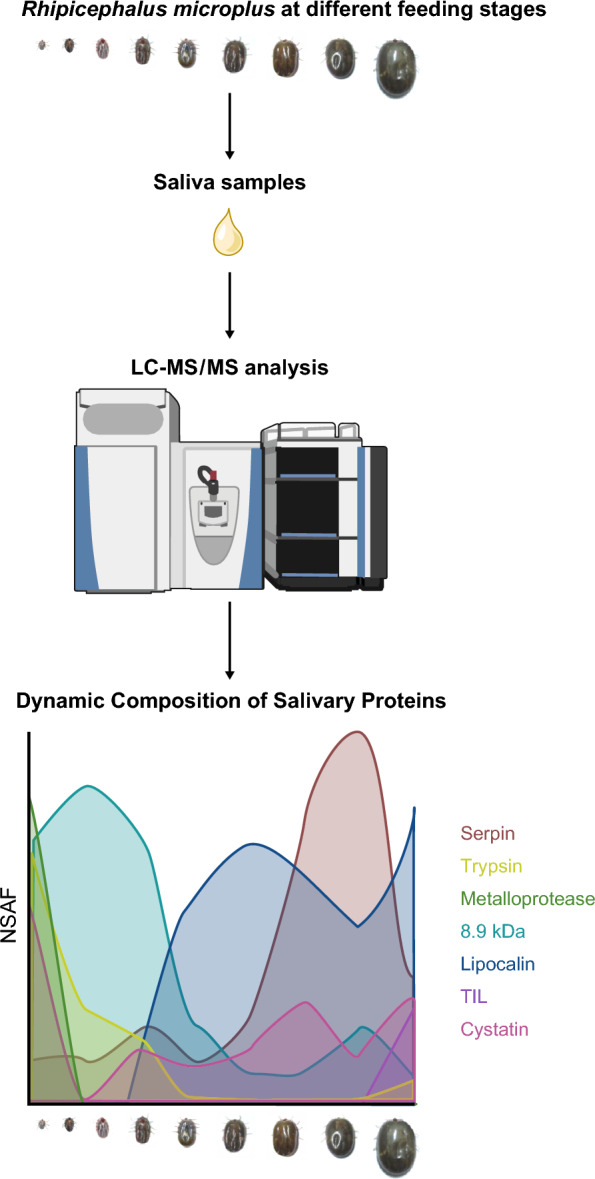

**Supplementary Information:**

The online version contains supplementary material available at 10.1186/s13071-024-06136-5.

## Background

Ticks are pool feeders that accomplish feeding by lacerating small blood vessels and ingesting the blood that flows from the feeding site. The hard tick feeding cycle lasts a few days and includes three phases: (i) the preparatory feeding phase, during which the tick attaches onto host's skin and creates the feeding lesion; (ii) the slow feeding phase, when the tick ingests moderate amounts of blood, begins to transmit pathogens, and grows new tissue to prepare itself for (iii) the rapid feeding phase, when the tick feeds until it reaches repletion [1]. This feeding style triggers several host-derived responses, and ticks face the problem of wound healing, which is achieved through four precisely and highly programmed phases: hemostasis, inflammation, cell proliferation and migration, and tissue remodeling. Together, these phases are barriers to acquiring a blood meal [2, 3]. To overcome these barriers, tick salivary glands have evolved a complex and sophisticated pharmacological armamentarium consisting of bioactive molecules to assist blood feeding [4].

While assisting the vector in feeding, tick saliva also modifies the site where pathogens are injected and, in many cases, facilitates the infection process [5]. Tick saliva contains a mixture of secretions from different salivary gland acini, making its composition variable and influenced by various factors [2]. It is evident that the kinetics and amount of saliva secretion differ considerably during the feeding process [6–9]. This effect is attributed to host and environmental stimuli, morphological and physiological alterations of salivary glands during the parasitic period, among other factors [2].

Transcriptomic and proteomic analyses of ticks have enabled the study of gene expression changes in different tick species and have been instrumental in understanding tick physiology and the biology of salivary glands and other tissues [10, 11]. Bioinformatic analyses of sequencing data are used to identify proteins and predict their function, providing a better understanding of how blood feeding and pathogen infection impact tick physiology. Although some progress has been made in recent years [11, 12], information on how tick saliva composition is modified during the tick feeding process is still scarce. For the cattle tick *Rhipicephalus microplus*, which is a one-host tick that feeds on bovines and is considered one of the most harmful cattle parasites in subtropical areas worldwide [13], our group pioneered the characterization of its saliva proteome by comparing saliva from partially and fully engorged females [9]. In the present study, we conducted a comprehensive analysis to evaluate changes in protein content profiles of *R. microplus* saliva in adult females throughout the entire feeding process. Uniquely, instead of grouping the different feeding stages based on partially and fully engorged ticks, we divided the ticks into nine groups based on their weight. Dividing ticks into groups by weight provides a better indicator of the physiological status of the tick feeding process [14]. The analyzed groups in this study represent the slow and rapid feeding phases of blood feeding. Mass spectrometry was used to identify differentially secreted proteins in tick saliva, and bioinformatic tools were employed to predict the structural and functional features of the salivary proteins. This work provides new insights into the plasticity of salivary protein secretion and the identification of compounds that may play a role in tick biology. Furthermore, this catalog of secreted salivary proteins serves as the basis for subsequent studies conducted by many other research groups.

## Methods

### Ticks and saliva collection

*Rhipicephalus microplus* ticks (Porto Alegre strain), free of pathogens such as *Babesia* spp. and *Anaplasma spp.,* were reared on Hereford calves (*Bos taurus taurus*) that were brought from a naturally tick-free area (Santa Vitória do Palmar, RS, Brazil; 33°32′2"S, 53°20′59"W) and maintained in individual boxes. Calves were infested with approximately 20,000 10-day-old larvae (from 1 g of *R. microplus* eggs), and after 21 days, adult females attached to the hosts were manually collected [15]. Adult females were collected, weighted, and grouped in nine groups (weight ± SD): group Rm-1 (34 ticks, weight 6.8 ± 1.1 mg); group Rm-2 (22 ticks, weight 15.8 ± 1.3 mg); group Rm-3 (16 ticks, weight 25.1 ± 1.3 mg); group Rm-4 (40 ticks, weight 35.0 ± 2.5 mg); group Rm-5 (25 ticks, weight 52.5 ± 2.8 mg); group Rm-6 (22 ticks, weight 88.0 ± 12.6 mg); group Rm-7 (16 ticks, weight 172.5 ± 16.1 mg); group Rm-8 (39 ticks, weight 270.2 ± 14.9 mg); and a group representing fully engorged females, group FEF (12 ticks, weight 347.5 ± 9.0 mg).

Ticks were rinsed with sterile water, dried on a paper towel, and placed dorsal side down on a glass slide containing tape. Salivation was induced by injecting 1–5 μl of 2% pilocarpine hydrochloride (in phosphate-buffered saline, pH 7.4) on the ventral side of the lower right coxa using a Hamilton syringe (Hamilton Co., Reno, NV, USA) [16]. Subsequently, after the injections, saliva was periodically collected using a Hamilton syringe (every 15 min over approximately 4 h). Protein concentration was determined using the bicinchoninic acid method (BCA Protein Assay, Pierce, Rockford, IL, USA) and stored at − 70 °C for later use.

### Protein digestion and sample preparation

The saliva of *R. microplus* ticks from each specific feeding group was digested in a solution with trypsin. First, the saliva was diluted in 8 M urea/0.1 M Tris, pH 8.5, reduced with 5 mM Tris(2-carboxyethyl) phosphine hydrochloride (TCEP, Sigma-Aldrich, St Louis, MO, USA) and alkylated with 25 mM iodoacetamide (Sigma-Aldrich). Proteins were digested overnight at 37 °C in 2 M urea/0.1 M Tris pH 8.5, 1 mM CaCl_2_ with trypsin (Promega) at a final ratio of 1:20 (enzyme:substrate). Digestion reactions, at a final concentration of 0.15 µg/ml, were quenched with formic acid (5% final concentration) and centrifuged to remove debris.

### Pre-columns and analytical columns

Reversed phase pre-columns were prepared by first creating a Kasil frit at one end of a deactivated 250 µm ID/360 µm OD capillary (Agilent Technologies, Santa Clara, CA, USA). Kasil frits were prepared by dipping 20 cm capillary in 300 µl Kasil 1624 (PQ Corporation, Malvern, PA, USA) and 100 µl formamide solution, curing at 100 °C for 3 h, and adjusting the length. Pre-columns were packed in house (John Yates III's laboratory at the Scripps Research Institute) with 2 cm of 5 µm ODS-AQ C18 (YMC America, INC., Allentown, PA, USA) particles from particle slurries in methanol. Analytical reversed-phase columns were fabricated by pulling a 100-µm ID/360 µm OD silica capillary (Molex Polymicro Technologies™, Austin, TX, USA) to a 5-µm ID tip. The same packing material was packed for a length of 20 cm directly behind the pulled tip. Reversed-phase pre-columns and analytical columns were connected using a zero-dead volume union (IDEX Corp., Upchurch Scientific, Oak Harbor, WA, USA).

### LC-MS/MS

Peptide mixtures were analyzed by nanoflow liquid chromatography mass spectrometry using an Easy NanoLC II and a Q Exactive mass spectrometer (Thermo Scientific, Waltham, MA, USA). Peptides eluted from the analytical column were electrosprayed directly into the mass spectrometer. Buffer A and B consisted of 5% acetonitrile/0.1% formic acid and 80% acetonitrile/0.1% formic acid, respectively. The flow rate was set to 400 nl/min. Saliva samples (1.5 µg per injection) were separated in 155-min chromatographic runs, as follows: a 1–10% gradient of buffer B in 10 min, a 10–40% gradient of buffer B in 100 min, a 40–50% gradient of buffer B in 10 min, and a 50–90% gradient of buffer B in 10 min. The column was held at 90% buffer B for 10 min, reduced to 1% buffer B, and re-equilibrated prior to the next injection.

The mass spectrometer was operated in a data-dependent mode, collecting a full MS scan from 400 to 1200 m/z at 70,000 resolution and an AGC target of 1 × 10^6^. The 10 most abundant ions per scan were selected for MS/MS at 17,500 resolution and AGC target of 2 × 10^5^ and an underfill ratio of 0.1%. Maximum fill times were 20 and 120 ms for MS and MS/MS scans, respectively, with dynamic exclusion of 15 s. Normalized collision energy was set to 25. Three technical replicates were used to analyze the protein extracts from tick saliva samples.

### Data analysis

Tandem mass spectra were extracted from Thermo RAW files using RawExtract 1.9.9.2 [17] and searched with ProLuCID [18] against a non-redundant database. The database consisted of an *R. microplus* database (22,009 entries) [19] concatenated with a *B. taurus* Uniprot reference database (23,868 entries) and reverse sequences of all entries. The searches were performed using the Integrated Proteomics Pipeline—IP2 (Integrated Proteomics Applications, Inc.). The search space included all fully tryptic and half-tryptic peptide candidates. Carbamidomethylation of cysteine was used as a static modification. The data were searched with a 50-ppm precursor ion tolerance and a 20-ppm fragment ion tolerance.

The validity of the peptide spectrum matches (PSMs) generated by ProLuCID was assessed using the Search Engine Processor (SEPro) module from the PatternLab for Proteomics platform [20]. Identifications were grouped by charge state and tryptic status, resulting in four distinct subgroups. For each group, ProLuCID XCorr, DeltaCN, DeltaMass, ZScore, the number of peaks matched, and secondary rank values were used to generate a Bayesian discriminating function. A cutoff score was established to accept a false discovery rate (FDR) of 1% based on the number of decoys. This procedure was independently performed on each data subset, resulting in a false-positive rate that was independent of tryptic status or charge state. Additionally, a minimum sequence length of six residues per peptide was required. The results were post-processed to only accept PSMs with < 10 ppm precursor mass error. Furthermore, only proteins identified in two out of the three technical replicates were considered for functional annotation.

### Protein functional annotation and classification

For annotation, we used an in-house program that scans a vocabulary of approximately 400 words and their order of appearance in the protein matches from BLASTp results, including their similarities and coverage [21]. Automated annotation of the tick proteins was based on matches to various databases, including Chelicerata from NCBI (https://www.ncbi.nlm.nih.gov/genbank/) and UniProt (https://www.ebi.ac.uk/uniprot/), UniProtKB (https://www.uniprot.org/help/uniprotkb), CDD, COG, KOG, PFAM, PRK, TIGR, SMART (ftp://ftp.ncbi.nih.gov/pub/mmdb/cdd/little_endian/), MEROPS (https://www.ebi.ac.uk/merops/), RefSeq invertebrate (https://ftp.ncbi.nlm.nih.gov/refseq/release/invertebrate/), and the TickSialoFam [11]. A database containing markers for extracellular vesicles was also used [22]. Signal peptide, furin cleavage, transmembrane, GPI anchors, and glycosylation site predictions were determined using software from the Center for Biological Sequence Analysis (https://www.cbs.dtu.dk/services/).

The identity to other tick proteins was evaluated by BLASTp using coding sequences extracted from tick genomes including *Dermacentor silvarum*, *Haemaphysalis longicornis*, *Hyalomma asiaticum*, *Ixodes persulcatus*, *I. scapularis*, *Rhipicephalus microplus*, and *R. sanguineus* [23].

### Graphical visualization

Normalized spectral abundance factors (NSAF) were used to represent relative abundance and secretion dynamics. Secretion dynamic plots were generated using the *ggplot2* library [24]. To verify whether the proteins identified in the different groups were organized in clusters, the NSAF values of the tick proteins were submitted to the CLICK algorithm of the Expander program [25]. For heatmaps, NSAF values were normalized by calculating the Z-score, and these values were used to generate heatmaps using the *heatmap2* function from the *ggplot2* library. Principal component analysis (PCA) was performed using NSAF values and the *prcomp* function from the *ggfortify* library [26].

### Structure prediction of salivary proteins

The Alphafold2 program [27] was used to predict the tertiary structures of *R. microplus* salivary proteins that were identified. The program was run locally on the NIH Biowulf cluster in a Linux environment using the monomer mode. The Dali program [28] was used to compare the Alphafold2 predictions to the structures available in the PDB database. The program was run locally in the NIH Biowulf cluster. Structures were visualized, and figures were generated using PyMol (PyMOL Molecular Graphics System, version 2.6.0a0, Schrödinger, LLC). The spreadsheet (Additional file 3: Table S3—sheet tab “AlphaFold”) has links to pdb files, which need programs that can open them. We suggest the use of ChimeraX, available online (https://www.cgl.ucsf.edu/chimerax/download.html).

### Identification of reciprocal best hits in other tick saliva proteomes

To identify reciprocal best hits with tick saliva proteins described in other studies, a BLASTp analysis was performed using pairwise analysis. A final cutoff value of 1e-6 and a coverage of at least 50% were selected [29]. Databases were retrieved from studies describing proteomic studies of saliva from *Ornithodoros moubata* males and females [30], *R. microplus* partially and fully engorged females [9], *H. longicornis* nymphs and adult females [8], *I. scapularis* and *Amblyomma americanum* adult females [7, 31], *I. scapularis* and *A. americanum* unfed adult females exposed to different vertebrate hosts [32], *Amblyomma sculptum* adult females [33], and *I. scapularis* nymphs infected and non-infected with *Borrelia burgdorferi* [34] and from cement from *A. americanum* [6] and *I. scapularis* [35].

### Alignment and sequence analysis

Protein sequence alignments were performed using Clustal W within the BioEdit 7.2.6.1 [36] and visualized with GeneDoc version 2.7 [37].

## Results and discussion

### An overview on *Rhipicephalus microplus* saliva proteome throughout the blood feeding

**Fig. 1:**
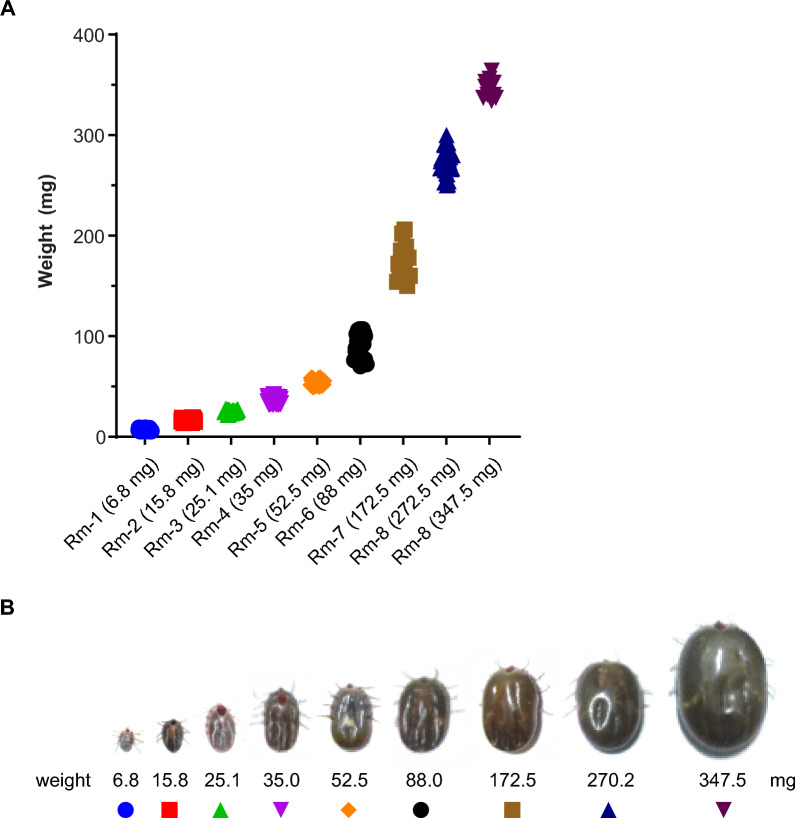
Group of *Rhipicephalus microplus* ticks collected through blood feeding. **A** Adult *R. microplus* females were collected, weighed, and divided into nine groups as follows (mean ± SD): (Rm-1) 6.8 ± 1.1 mg; (Rm-2) 15.8 ± 1.3 mg; (Rm-3) 25.1 ± 1.3 mg; (Rm-4) 35.0 ± 2.5 mg; (Rm-5) 52.5 ± 2.8 mg; (Rm-6) 88.0 ± 12.6 mg; (Rm-7) 172.5 ± 16.1 mg; (Rm-8) 270.2 ± 14.9 mg; and a group representing fully engorged females (FEF) weighing 347.5 ± 9.0 mg. A graphical representation demonstrates the grouping of ticks according to weight and their relationship with the feeding period. This graph illustrates the initial slow-feeding phase (from Rm-1 to Rm-6), followed by the rapid-feeding phase (from Rm-6 to FEF). **B** From left to right, a series of images showing a representative tick from each group, spanning from Rm-1 to FEF

The *R. microplus* feeding cycle spans several days and consists of three distinct phases [1, 38]. The present study complements a pioneering study developed by our group when we compared the proteomic profile of saliva from partially and fully engorged *R. microplus* females [9]. Ticks were fed on calves, and adult females were collected, weighed, and divided into nine groups. Uniquely, instead of grouping the different feeding stages by days of feeding, we grouped the ticks by their weight, as it is a better indicator of the physiological status of the tick feeding process [19, 39]. Ticks in these engorgement stages represent feeding phases that adult ticks pass through during the blood-feeding process, including representatives from the slow feeding phase, rapid feeding phase, and fully engorged ticks (Fig. 1).

Pilocarpine-induced saliva was successfully harvested from these ticks, and protein identification was performed by LC–MS/MS. The generated raw data from the shotgun proteomic approach were used to query the *R. microplus* de novo transcriptome assembly using PatternLab software, which allows data normalization by the normalized spectral abundance factor (NSAF) approach. The search of extracted tandem mass spectra against the tick and host protein databases produced hits to 346 tick-derived proteins (Additional file 1: Table S1) and 74 host-derived proteins (Additional file 2: Table S2). When subjected to validity analysis in the PatternLab for Proteomics platform, 284 of the 346 tick-derived proteins were considered true proteins since they were detected in a minimum of two out of the three runs (Additional file 1: Table S1—sheet tab “(Additional file 1: Table S1A”), while the remaining 62 proteins detected in only one of the three runs were considered low confidence and were not further analyzed (Additional file 1: Table S1—sheet tab “Additional file 1: Table S1B”). Of the 74 host-derived proteins detected in *R. microplus* saliva, 47 met the criteria for authentication (Additional file 2: Table S2—sheet tab “Table S2A”), and the remaining 27 were not further analyzed (Additional file 2: Table S2—sheet tab “Table S2B”). The 284 tick-derived proteins were annotated and classified into 22 different functional classes (Additional file 3: Table S3).

### Insight into the sialome switch of *Rhipicephalus microplus*

During the process of feeding, the salivary glands play an essential role by secreting a unique collection of molecules, forming a complex and ever-changing cocktail. This mixture exhibits a dynamic composition, with the amounts of each protein constantly varying in response to various factors, including developmental stage, blood meal uptake, time/duration of the feeding process, the host where ticks are feeding, and whether they are infected or not with different pathogens [5, 32, 40, 41], the phenomenon called "sialome switching” [42, 43].

**Fig. 2:**
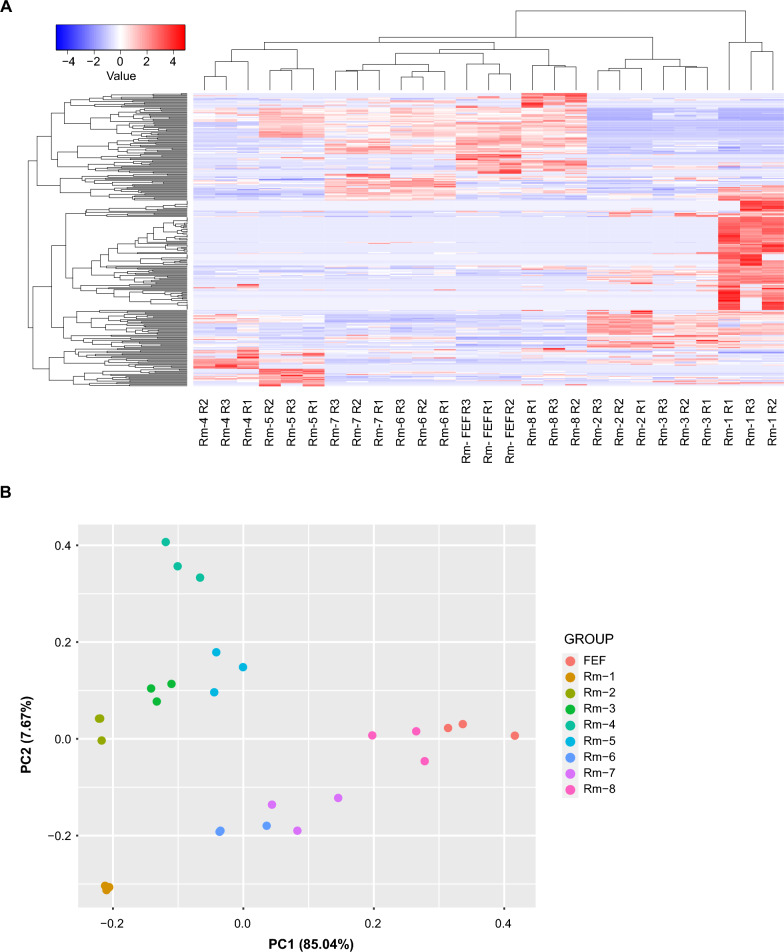
An overview of *Rhipicephalus microplus* saliva proteome throughout blood feeding. **A** Heat map of *R. microplus* tick saliva proteome throughout blood feeding. This heat map illustrates the changes in the *R. microplus* tick saliva proteome throughout blood feeding. Blue indicates downregulation, while red indicates upregulation. **B** Principal component analysis (PCA) of *R. microplus* tick saliva proteome throughout blood feeding

**Fig. 3:**
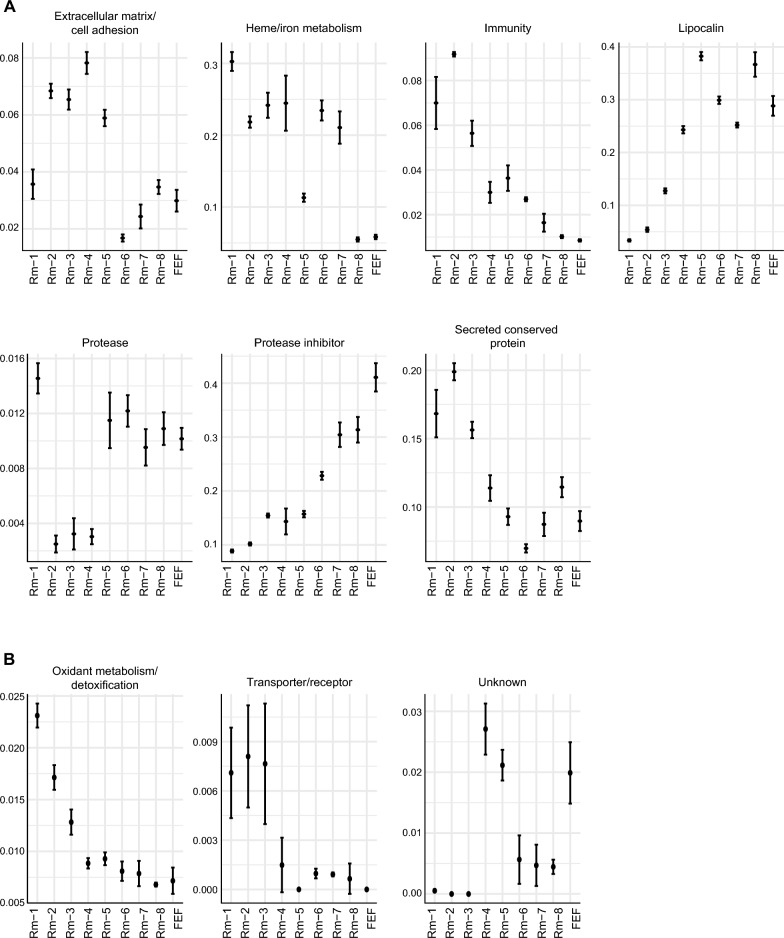

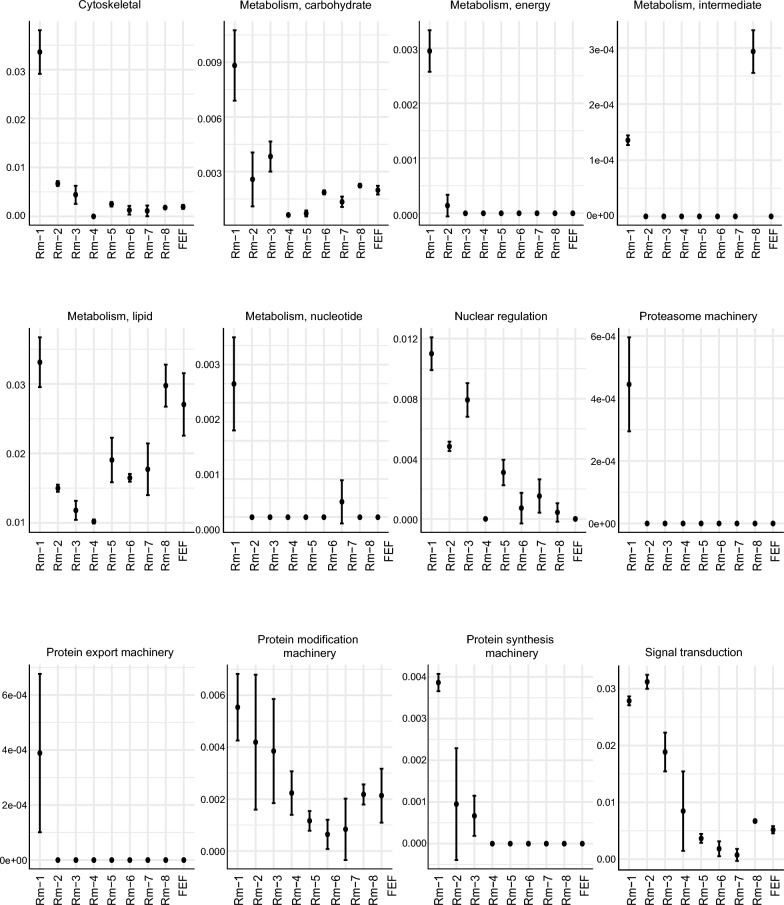
Expression patterns of the functional classes identified in the *Rhipicephalus microplus* saliva proteome throughout blood feeding. Each data point represents the average NSAF value for each functional group, with error bars denoting the standard error. **A** Represents classes of proteins with higher abundance. **B** Represents classes of proteins with lower abundance

To gain insight into the broad relationships of secretion dynamics of tick proteins with tick feeding processes, the Z-score was used to represent NSAF values, which were visualized on a heatmap. The heatmap displayed how dynamic the expression of salivary proteins during blood feeding is (Fig. 2A). Indeed, the PCA plot (Fig. 2B) showed remarkable clustering of the replicates and distinct proteomic profiles among all groups, revealing the phenomenon of "sialome switching.” The 284 tick-derived proteins were annotated and classified into 22 different functional classes. Notably, the saliva of *R. microplus* contained a variety of highly abundant proteins, some of which play important roles in blood feeding. These proteins include those associated with heme/iron metabolism, lipocalins, secreted conserved proteins, protease inhibitors, proteases, immunity, and the extracellular matrix (Fig. 3A). The other 15 classes are less abundant; little is known about their role in tick feeding (Fig. 3B), and they will not be discussed in detail.

#### Hemelipoproteins

As previously demonstrated [9], *R. microplus* saliva contains a high concentration of hemelipoproteins, with a specific focus on the HeLp protein [9, 44, 45]. HeLp possesses the capability of heme binding, in addition to cholesterol, phospholipids, and free fatty acids [44, 45]. Hemelipoproteins, as classified in the heme/iron metabolism plot in Fig. 3, are one of the most abundant proteins found in the saliva of *R. microplus* during the slow and rapid feeding phases (Rm-1 to Rm-7). However, their presence in saliva decreases as the feeding process progresses towards the later stages of engorgement (Rm-8 and FEF). Although only six proteins belonging to this class have been identified, these six proteins contribute to most spectral counts observed in tick saliva (Additional file 1: Table S1 and Additional file 3: Table S3).

Given the primary localization of HeLp in the hemolymph of ticks [44, 45], its presence in the saliva could be attributed to the incorporation of hemolymph components by the salivary glands. However, research focusing on the transcriptional profile and protein localization of these hemelipoproteins in the salivary glands of adult and unfed ticks from various species has suggested that they may have diverse functions during blood feeding [46, 47]. Furthermore, the identification of these proteins in the saliva of other tick species demonstrates their conservation across different ticks [7, 8, 31, 48–50]. These findings suggest a crucial role for HeLp in tick feeding. HeLp possesses the capability to bind eight heme molecules [44, 45]. Consequently, its inoculation into the host can reduce the concentration of free heme at the feeding site, effectively preventing inflammation [51]. Additionally, this protein can bind to other molecules, including cholesterol, phospholipids, and free fatty acids [44, 45], and may be molecules yet unknow. It is plausible that these compounds could be carried at the feeding site bound to HeLp, potentially carrying small pharmacologically active molecules from salivary origin. Similarly, as suggested for the sequestration of heme at the feeding site, HeLp acting as a kratagonist of other small host-derived molecules [52] at the feeding site cannot be ruled out. Under these circumstances, hemelipoproteins may serve as heme transporters and/or kratagonists during the onset of hemoglobin digestion in the midgut. The role of these proteins in the interaction between ticks and their hosts remains largely unknown. Given their significant presence in tick saliva, conducting studies to elucidate their functions during the blood meal is crucial.

#### Lipocalins

Lipocalins are also highly abundant in tick saliva, with their levels increasing during the feeding process and reaching a peak in the saliva of ticks from the group Rm-5, remaining steady until the end of the feeding process (Fig. 3A). In total, *R. microplus* ticks secrete a minimum of 59 different lipocalins in their saliva. This observation is in accordance with studies describing the high abundance of lipocalins in the saliva of different tick species [7–9, 31, 48, 53].

Lipocalins are proteins composed of approximately 200 amino acids, known for their compact folding into a β-barrel shape. Within this structure, a central pocket capable of binding small hydrophobic molecules exists. In ticks, lipocalins play a crucial role as kratagonists, effectively scavenging histamine, serotonin, leukotriene B4, leukotriene C4, thromboxane A2, and cholesterol. By modulating the host's hemostasis, inflammation, and other immune responses, these lipocalins facilitate blood feeding [54]. When subjected to a scan using the Pfam database, it was revealed that, out of the 59 identified and classified as lipocalins, at least 43 possess the tick histamine-binding domain (PF02098) (Additional file 3: Table S3). Despite the known kratagonist activities of tick lipocalins, the biochemical characterization of lipocalins from *R. microplus* is lacking, thus hindering the identification of their primary ligands.

#### Proteases

A total of 25 proteases belonging to various families were identified in the saliva of *R. microplus*. These proteases include 12 metalloproteases, 6 serine proteases, 5 asparagine proteases, and 2 cysteine proteases. Searching the MEROPS database showed that the 12 metalloproteases can be classified into families M12B (*n* = 9), M17 (*n* = 2), and M13 (*n* = 1), while the asparagine and cysteine proteases are categorized into families A1 and C1, respectively. The serine proteases identified in this study were assigned to families S01 (*n* = 3) and S10 (*n* = 3) (Additional file 3: Table S3).

Notably, the saliva from ticks in the Rm-1 group contained the highest abundance of proteases. Their abundance decreased from groups Rm-2 to Rm-4, increased in group Rm-5, and remained relatively steady until the end of the feeding period (Fig. 3A). Examining the expression dynamics of proteases according to their class shows that metalloproteases, including members from M12B, M17, and M13 families, reach their peak at the start of feeding exclusively in the Rm-1 group. Their levels sharply decline in the Rm-2 group and remain consistently low until the end of the feeding period (Additional file 4: Fig. S1). The predominance of family M12B metalloproteases secreted by *R. microplus* at the early stages of blood feeding indicates their significance in tick feeding physiology. Tick metalloproteases have activities that promote tick feeding [55], and their blockage by silencing or immunization has an impact on tick feeding [56–58]

Since the discovery of the metalloproteoid family [59], we have decided to reanalyze the metalloprotease family to determine if each sequence contains the Zn-binding domain. Thus, we conducted a search and manually checked for the motif H-E-x(2)-H–x(2)-G-x(2)-H [59]. Among the 12 proteins identified within the M12B, M13, and M17 families, Rm-1513, Rm-156308, Rm-18262, Rm-24243, and Rm-2425 were found not to possess the Zn-binding domain and could be classified as part of the metalloproteoid family (Additional file 5: Fig. S2). Metalloproteases exhibit a wide range of substrate specificity, including coagulation factors, platelet membrane receptors, and von Willebrand factor [60]. It is possible that tick metalloproteoids function by specifically binding to host proteins without hydrolyzing them, but potentially inhibit their function, thereby acting as pseudoenzymes or kratagonists [59].

The proteases belonging to the S01 family exhibit a peak within the Rm-1 group, followed by a gradual reduction through the Rm-5 group. However, their levels rise again in the Rm-8 group before declining once more towards the end of the blood feeding (Additional file 4: Fig. S1). On the other hand, the proteases affiliated with the S10 family demonstrate a peak between the Rm-4 and Rm-6 groups. Subsequently, their levels decline and then remain steady until the end of the blood feeding (Additional file 4: Fig. S1). Upon examining the primary and tertiary structure of the members within the S01 family, it became apparent that Rm-77083 and Rm-163513 exhibit the characteristic folding pattern of serine proteases, featuring the catalytic triad comprising Asp, His, and Ser. However, notably, Rm-2605, despite being annotated in this family, deviates from this pattern by substituting Ser in the catalytic triad with Gly (Additional file 6: Fig. S3) [61]. The presence of trypsin-like proteases in tick saliva, especially members within the S01 family, may be a strategy to interfere with host inflammation and blood clotting similarly to what has been reported for *I. scapularis* saliva [62].

Proteases associated with the C01 family display a peak at the beginning of the feeding period (Rm-1 to Rm-3), which is subsequently followed by a decrease in groups Rm-4 and Rm-5. Their levels then rise again in the Rm-6 and Rm-7 groups before declining once more towards the end of the blood meal (Additional file 1: Fig. S1). On the other hand, the proteases associated with the A01 family are not expressed at the beginning of feeding; instead, their presence in the saliva gradually increases from Rm-4 to Rm-5 and remains constant until the end of feeding (Additional file 1: Fig. S1). During the examination of the primary and tertiary structure of the members within the C01 family, it became evident that both proteins within this family exhibit the characteristic folding pattern of cysteine proteases, including the catalytic dyad comprising Cys and His, as depicted in Additional file 7: Fig. S4. Most members of the C01 and A01 families have been well characterized as important players during blood meal digestion in the midgut [63, 64] and vitellin digestion during embryogenesis [65, 66]. However, their role in the saliva remains unclear. Further characterization of the tick saliva proteases identified in this study holds great promise for gaining valuable insights into their crucial role in the blood feeding process.

#### Protease inhibitors

Regarding protease inhibitors, in a similar fashion to the observation of proteases, a total of 26 protease inhibitors were identified in the saliva of *R. microplus* (Additional file 3: Table S3). However, they exhibited a higher abundance compared to the proteases (Fig. 3A). Based on the literature and information from the MEROPS database, these protease inhibitors were classified into eight families: serpins (*n* = 9), which include RmS-7, RmS-15, and various isoforms of RmS-3, RmS-6, and RmS-17 [9], trypsin-inhibitor like (TIL) (*n* = 5), Kunitz type (*n* = 3), cystatins (*n* = 3), BmSEI-like (*n* = 3), thyropins (*n* = 2), and Kazal type (*n* = 1).

Serpin is a class of protease inhibitors which regulates the activity of serine proteases by forming irreversible covalent complexes with them [67]. Serpins were already described in the saliva of ticks and specifically play a crucial role in tick-host interactions by modulating the host responses against tick feeding [68]. The reactive center loop (RCL) of serpins consists of a flexible stretch of 21 amino acid residues, serving as a pseudo-substrate for the target protease. The P1 residue within the RCL plays a crucial role in determining the specificity of a serpin for a particular protease [69]. Tick serpins identified in this study exhibit different P1 residues, including basic side chains (Lys for RmS-17 and Arg for RmS-6 and RmS-15), polar side chains (Ser for RmS-7), and hydrophobic side chains (Leu for RmS-3) [9], potentially suggesting these serpins target different proteases during blood feeding. The serpins identified in the saliva of *R. microplus* exhibit a basal content at the initiation of feeding (Rm-1 to Rm-5), which is subsequently followed by an increase in groups Rm-6 and Rm-7. However, their levels decrease again in group Rm-8 and rise once more in group FEF (Additional file 8: Fig. S5). Serpins RmS-3, RmS-6, RmS-15, and RmS-17 have been biochemically characterized and possess anticoagulant and immunomodulatory properties, suggesting the potential for these serpins to target host responses during blood feeding [70–72].

In arthropods, most trypsin inhibitor-like proteins (TILs) are composed of a single domain featuring two roughly perpendicular β-sheets, turns, and a long-exposed loop that encompasses the protease binding site (RCL). The protein is reinforced by five disulfide bridges, which enhance the stability of both the protein scaffold and the RCL. Furthermore, the RCL demonstrates similarities to those observed in other canonical serine protease inhibitors [73]. In this study, three TILs (Rm-10457, Rm-18594, and Rm-32449) were found to have a single domain, while two TILs (Rm-53166 and Rm-53167) exhibited two domains separated by a flexible loop. TILs with a single domain featured Leu (Rm-10457) and Ala residues (Rm-18594 and Rm-32449) at the P1 position. Interestingly, TILs with two domains have Tyr residues at the N-terminal domain and Ala residues at the C-terminal domain as their respective P1 residues (Additional file 9: Fig. S6), suggesting the potential to target two different proteases. The presence of proteins of this family in saliva has a peak at the beginning feeding (Rm-1 to Rm-3), which is then followed by a decrease in expression in groups Rm-4 to Rm-6. However, their levels rise again in group Rm-7 and continue to increase until the end of feeding (Additional file 8: Fig. S5). Only a few TIL proteins have been functionally characterized in ticks. One such protein, ixodidin, which we identified here (Rm-10457), was previously isolated from *R. microplus* hemocytes. It exhibits antimicrobial activity and the ability to inhibit elastase and chymotrypsin [74]. Another *R. microplus* protein containing a TIL domain, named BmSI-7, was also identified in our study (Rm-32449). BmSI-7 displays inhibitory activities against subtilisin A and human neutrophil elastase, suggesting a potential role in modulating inflammation at the tick feeding site on the host's skin [75]. Notably, antibodies against BmSI-7 have been shown to affect female ticks' reproductive parameters in an artificial tick feeding assay [76].

Cystatins, which act as cysteine protease inhibitors, are present in various organisms and are classified into three subfamilies based on their primary structure. In ticks, both intracellular (type 1) and secretory (type 2) cystatins have been identified and characterized. Here, three type 2 cystatins have been identified: Rmcystatin-2, BrBmcys2a, and BrBmcys2b. These cystatins inhibit host cathepsin B, cathepsin C, and cathepsin L, potentially regulating the host's immune response [77–79]. Similarly, members of the thyropin protein family are characterized by the presence of thyroglobulin type-1 domain repeats. A well-characterized thyropin protein was initially described in a sea anemone [80] and has been demonstrated to inhibit both cysteine and cation-dependent proteases, including cathepsin L, cathepsin S, papain, and cruzipain [80]. Despite this, the functional characterization of thyropins in ticks is currently lacking. However, the presence of these proteins in tick saliva strongly suggests their potential involvement in modulating host immune responses [81]. When analyzing the saliva profiles of these two inhibitor families, a distinct difference becomes evident. Cystatins exhibit nearly constant basal levels throughout the entire blood meal, whereas thyropins display a peak at the beginning of feeding (Rm-1 to Rm-4) followed by a gradual decline to sustained levels until the end of the meal (Additional file 8: Fig. S5).

Members of the Kunitz-type family have been extensively studied in ticks as inhibitors of numerous serine proteases, affecting blood coagulation and immune responses [82, 83]. In this study, three proteins containing Kunitz-type domains were identified. Interestingly, only one protein (Rm-51801) displayed a structure resembling the canonical Kunitz-type inhibitors, whereas the other two (Rm-4223 and Rm-770) exhibited non-canonical structures (Additional file 3: Table S3—sheet tab “AlphaFold”). Their presence in saliva peaks in the Rm-1 group drops to zero in the Rm-2 to Rm-5 groups, and then rises again in the Rm-6 group, remaining relatively constant until the end of the blood meal (Additional file 8: Fig. S5).

A single Kazal domain-containing protein was identified in the saliva, with exclusive expression observed in the Rm-1 group (Additional file 8: Fig. S5). Despite the previous classification of BmSEI as a protease inhibitor, recent studies have revealed that these proteins lack inhibitory activity but possess antimicrobial properties [76]. Notably, the structure of BmSEI (Rm-928) reveals the presence of alpha helices (Additional file 3: Table S3—sheet tab “AlphaFold”), which are characteristic of antimicrobial peptides [84].

#### Extracellular matrix/cell adhesion

Extracellular matrix/cell adhesion proteins encompass a large group of proteins that play roles in both structural and non-structural functions, contributing to the organization of cells and tissues [85]. In *R. microplus* saliva, the number of extracellular matrix proteins is elevated during the initial phases of blood feeding and decreases upon the completion of the process (Fig. 3A). Proteins within this class are represented by tick cement proteins such as glycine-rich proteins, followed by alanine- and proline-rich proteins, as well as laminin. Glycine-rich proteins have been implicated in the formation of the cement cone [35], and their heightened presence during the initial phases of blood feeding may be linked to the ticks' need to establish a secure attachment by means of the cement cone.

#### Secreted conserved proteins

Multiple proteomic and transcriptomic analyses of tick salivary glands have revealed the presence of numerous proteins that do not exhibit similarity to proteins found in other species within the public repositories. However, similar proteins have been identified in the saliva of various tick species and are referred to as secreted conserved proteins [11]. These proteins show the highest abundance in the initial saliva samples (groups Rm-1 to Rm-2) and gradually decrease towards the later samples (groups Rm-3 to FEF) (Fig. 3). These functionally unknown proteins comprise a significant fraction of the saliva of different tick species, suggesting a key biological role in tick biology. Further analyses using the TickSialoFam database have classified most of the proteins in these categories as follows: secreted conserved protein, secreted protein 28 kDa, secreted protein 13–14 kDa, secreted protein 23–24 kDa, 8.9 kDa family member, antigen 5/SCP domain-containing protein, tick salivary cytotoxin, ixodegrin, Mys-25–299, Mys-30–60, salivary protein one-of-each family, and salp15/ixostatin (Additional file 3:Table S3).

The 8.9-kDa family is exclusively found in hard ticks and exhibits a highly variable structure, with most forms containing one or two domains [59]. The only established function of the 8.9-kDa family is the inhibition of complement by binding to component C5 and preventing its activation, exemplified by the protein CirpT1 from *Rhipicephalus pulchellus* [86]. Using the crystal structure of CirpT1 complexed with C5 alone, a specific block of eight residues in the interaction interface was identified [59]. Among the five proteins classified within the 8.9-kDa family, Rm-3247 is the sole protein displaying this block of residues and appears to be the homolog of CirpT1 in *R. microplus* (Additional file 10: Fig. S7). Furthermore, the presence of proteins within the 8.9-kDa family exhibits a peak at the onset of the feeding period (Rm-1 to Rm-3), followed by a decline in group Rm-4, remaining relatively stable until the completion of the blood meal (Additional file 11: Fig. S8).

Proteins classified as cytotoxins are related to bacterial pore-forming proteins. The presence of these proteins in ticks suggests they were acquired by horizontal transfer [59], as was the case with the DAE antimicrobial proteins [87]. Nine of ten proteins annotated as cytotoxin are relatively more abundant at Rm-1 (Additional file 11: Fig. S8).

Ixostatin was the name given to a group of cysteine-rich protein sequences found in the sialotranscriptome of *Ixodes pacificus*. These sequences displayed remarkable similarities to the cysteine-rich domain of ADAMTS-4 (aggrecanase). The vast majority of ixostatins are found in the *Ixodes* genus, but a few sequences are also found in metastriate ticks. We have identified two sequences similar to Salp15 (Rm-19629 and Rm-66113), a protein isolated from *I. scapularis*, which inhibits CD4 + T cell activation [88].

The 13-kDa basic family is a small secretory family found in prostriate and metastriate ticks. This family is highly conserved among tick species and is characterized by six conserved cysteines. Alphafold2 predicts a compact globular structure formed by six alpha-helices. All structures present intact disulfide bonds, and the Dali search algorithm identified structural homologs among members of the odorant-binding, pheromone-binding, and venom 2 allergen families [59]. Odorant-binding proteins have not yet been found in ticks as prominent ligand scavengers involved in tick-host interactions, as this function is generally performed by lipocalins; however, a kratagonist function cannot be ruled out.

#### Immunity

The tick immune response is primarily based on innate immunity, with many compounds and mechanisms conserved across various species. While the hemolymph is the primary site of defense mechanisms in insects [89], the salivary glands express various immune response compounds that are secreted into the saliva [90, 91]. Consequently, in the saliva, numerous molecules with potential tick immunity-related activities have been detected. Specifically, proteins related to the immunity response are represented by microplusin and microplusin-like proteins, Toll-like receptors, alpha-2-macroglobulin-like proteins, evasins, ML domain-containing proteins, calreticulins, and DA P-36.

Microplusin is an antimicrobial peptide active against bacteria and fungi present in the tick *R. microplus* [92, 93] and, with a homologous peptide, in *Amblyomma hebraeum* [94]. The structure and biological function of microplusin have been characterized in the tick's hemolymph, ovaries, and eggs [92, 93]. The role of antimicrobial peptides in tick saliva may be associated with the prevention of microbial proliferation at the tick-feeding site.

The Toll-like pathway was originally discovered in *Drosophila* [95]. This highly conserved pathway presents in both invertebrates and vertebrates. It detects pathogen-associated molecular patterns (PAMPs), activating the immune response against various pathogens [96]. In ticks, the Toll-like system is not fully characterized, but the expression of its components is modulated by pathogen infection [89]. The precise nature of the presence of Toll-like compounds in saliva, whether they have a biological function or are a result of salivary gland degeneration, remains unclear. An interesting soluble form of Toll-like receptor displaying immunoregulatory functions has been found in human saliva [97].

Proteins with immunosuppressive activity clustered in the P36 family are immunosuppressive proteins identified in *Dermacentor andersoni* (DAP36) [98], *H. longicornis* (HL-p36) [99], *Rhipicephalus haemaphysaloides* (RH36) [100], and other ticks [101]. The suppressive activity was characterized in *D. andersoni* [102] and *H. longicornis* salivary glands [103]. Interestingly, in *R. microplus*, the removal of a *Coxiella*-like endosymbiont reduces the expression of the P36 protein, suggesting a complex host-microbiota-tick interaction [104].

Calreticulin is a Ca^2+^ binding protein with multifunctional properties. Initial studies showed it to be a molecular chaperone associated with the endoplasmic reticulum [105]. However, several studies have consistently shown that calreticulin has other roles as an intra- or extracellular protein [106], including in tick saliva [107–109]. Furthermore, studies have suggested a role for calreticulin as a potential biomarker of tick exposure in humans [110, 111] or an antigen for vaccination [112]. As a protein with immunomodulatory properties, it has been observed that calreticulin from various parasites, including *Trypanosoma cruzi* [113] and *Hemonchus contortus* [114], can inhibit the activation of the complement pathway and the blood clotting system. In contrast, the biological function of calreticulin in tick saliva remains unknown since these activities were not observed in *A. americanum* and *R. microplus* saliva [108, 109].

### An insight into the core proteins related to different phases of feeding in *Rhipicephalus microplus*

**Fig. 4:**
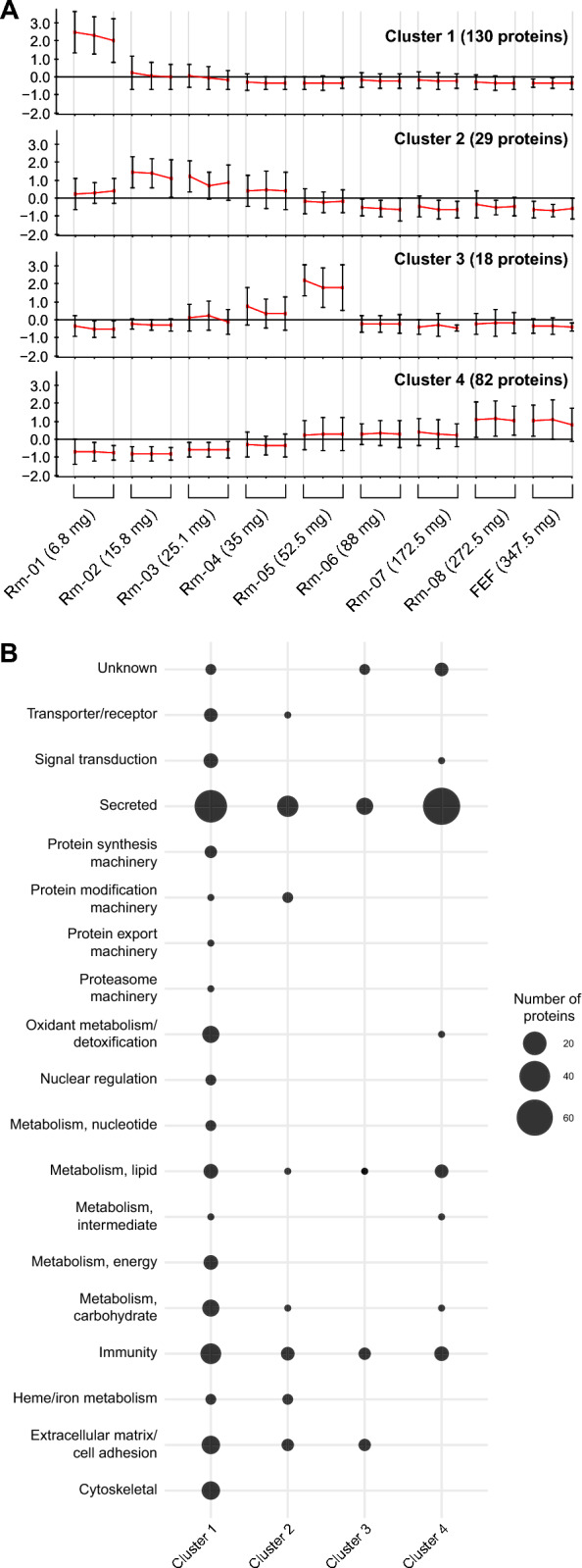
Unsupervised clustering of proteins identified in the *Rhipicephalus microplus* saliva proteome throughout blood feeding. **A** The patterns were identified through clustering analysis using the CLICK algorithm. The x-axis represents the tick groups, the y-axis represents log2-based ratios standardized expression levels of cluster groups, and error bars represent the standard error. Each cluster is presented with triplicate samples. In parentheses, the number of proteins identified in each cluster is shown. **B** The frequency of the functional classes of proteins identified within each cluster is represented by the size of each sphere

To determine whether proteins were organized in clusters associated with adult ticks at different feeding stages, we subjected the normalized NSAF values to the CLICK algorithm within the expander program, which identified four protein clusters (Fig. 4).

Cluster 1 consisted of 130 proteins, primarily found in the Rm-1 group, representing the early stage of feeding. These proteins belong to various classes, including "Cytoskeletal," "Extracellular matrix/cell adhesion" (including glycine-rich proteins), "Heme/iron metabolism" (including the heme-binding protein HeLp), "Immunity" (including antimicrobial microplusin), "Secreted-lipocalin," "Metabolism" (carbohydrate, lipid, and energy), "Oxidant metabolism/detoxification," "Secreted-protease" (including mainly proteases from the M12B family), "Secreted-protease inhibitor" (including serpins RmS-7 and RmS-15), and "Secreted-secreted conserved protein" (including cytotoxins and a potential inhibitor of complement from the 8.9-kDa family) (Fig. 4b and Additional file 3: Table S3).

Cluster 2 included 29 proteins, which were predominantly present in groups Rm-2 and Rm-3. Proteins within this cluster include "Extracellular matrix/cell adhesion" (including mucin and glycine-rich protein), “Secreted-secreted conserved protein” (including 8.9-kDa family, ixodegrin, 23–24 kDa), "Immunity" (including microplusin-like and Toll-like), and "Secreted-lipocalin" (Fig. 4b and Additional file 3: Table S3).

Cluster 3 contained 18 proteins, mainly associated with the Rm-5 group. Proteins within this cluster include "Extracellular matrix/cell adhesion" (including mucin and glycine-rich protein), “Secreted-secreted conserved protein” (including 13 – 14 kDa, 23–24 kDa, and unknow), "Immunity" (including defensin and ML domain-containing protein), and "Secreted-lipocalin" (Fig. 4b and Additional file 3: Table S3).

Cluster 4 comprised 82 proteins, with their highest presence observed as ticks approached the end of feeding, as represented by groups Rm-8 and FEF (Fig. 4). These proteins belong to various classes, including "Metabolism" (carbohydrate, lipid, and intermediate), "Immunity" (including antimicrobial microplusin, evasin, ML domain-containing protein, and immunoglobulin G-binding protein), "Secreted-lipocalin," "Secreted-protease" (including mainly proteases from the A01, S01, and S10 family), "Secreted-protease inhibitor" (including cystatin, BmSEI-like, serpins RmS-3 and RmS-6, and TIL), and "Secreted-secreted conserved protein" (including 23–24 kDa, Salp15-like, and unknown) (Fig. 4b and Additional file 3: Table S3).

These results confirm the presence of a 'sialome switch' within *R. microplus*, facilitating the identification of proteins with similar expression patterns. Proteins presented within these clusters may represent the core of proteins needed at different phases of blood feeding.

### An insight into the core proteins related to the sialome of different tick species

The identification of proteins in tick saliva and alterations in the saliva composition during the feeding process have been analyzed in various tick species. This includes proteome analysis of *O. moubata* males and females [30, 48], engorged females of* R. sanguineus* [49], partially and fully engorged female *R. microplus* [9], nymph and adult females of *H. longicornis* [8], and *A. sculptum* adult females [33]. Other studies have also performed analyses of saliva at different moments during tick feeding. In *I. scapularis* and *A. americanum*, saliva was analyzed at different day intervals [7, 31]. Additionally, the variation in saliva composition was analyzed in unfed *I. scapularis* and *A. americanum* ticks exposed to different hosts [32] and, in *I. scapularis*, nymphs infected and non-infected with *B. burgdorferi* [34].

To identify reciprocal best hits among tick saliva proteins described in the aforementioned studies, a BLASTp analysis was performed. We selected a final cutoff value of 1e^−6^ and required a coverage of at least 50%. In addition to the datasets of tick salivary proteins mentioned above, we included datasets of proteins identified in the cement of *A. americanum* [6] and *I. scapularis* [35].

**Fig. 5:**
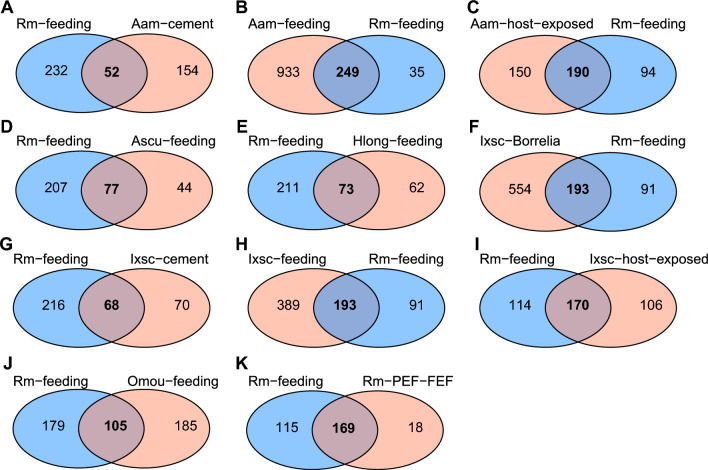
Venn diagram showing the number of shared orthologs in the *Rhipicephalus microplus* saliva proteome based on reciprocal best hits. The comparison is made with proteins identified in saliva of other tick species, including **A** *Amblyomma americanum* tick cement cone [6], **B** *A. americanum* saliva collected every 24 h for 8 days [7], **C** *A. americanum* saliva of tick exposed to different hosts [32], **D** *A. sculptum* saliva collected from unfed and partially fed ticks [33], **E** *Haemaphysalis longicornis* saliva collected from nymph and adult females [8], **F** *Ixodes scapularis* saliva collected from *Borrelia burgdorferi*-infected nymphs [34], **G** *Ixodes scapularis* tick cement cone [35], **H** *I. scapularis* tick saliva collected every 24 h for 5 days [31], **I** *I. scapularis* saliva of tick exposed to different hosts [32], **J** *Ornithodoros moubata* female and male saliva [30], and **K** *R. microplus* partially engorged female and fully engorged female saliva [9]

Of the 284 proteins identified in this study, 267 have reciprocal best hits with salivary proteins identified in other tick species (Fig. 5 and Additional file 3: Table S3—sheet tab “Best-reciprocal”). These data suggest that ticks utilize a core set of functionally similar proteins that can regulate key host defense pathways to successfully feed. Functional classes conserved in the saliva of different tick species include hemelipoproteins, lipocalins, metalloproteases, protease inhibitors (serpins, cystatins, and TIL), and secreted conserved proteins, among others. While the functional roles of most of these salivary proteins remain to be determined, available evidence indicates that some of these proteins may regulate important tick feeding pathways, with potential variations among different tick species. For instance, the complement inhibitor CirpT1 is a member of the 8.9-kDa family and inhibits complement by binding to component C5, preventing its activation [86]. Best reciprocal hits showed these proteins are present in the saliva of most tick species. Interestingly, only homologs from *R. microplus*, *A. americanum*, and *A. sculptum* display the block of eight residues in the interaction interface of CirtT1 with the C5 protein (Additional file 12: Fig. S9), suggesting similar proteins in different tick species may act by inhibiting complement. Proteins identified in the saliva of *I. scapularis* lack these residues. In fact, *I. scapularis* has evolved with Salp20-like proteins that inhibit exclusively the alternative pathway of complement [115, 116]. This may represent that, although similar functional groups of salivary proteins are shared among different tick species, different strategies are used by metastriate and prostriate ticks to overcome host responses during blood feeding.

### An insight into the host-derived proteins in the sialome of *Rhipicephalus microplus*

The presence of host-derived proteins or fragments of host-derived proteins was already described in several species as a true component of tick saliva and not as a contamination due to saliva harvesting procedures [9, 48, 49, 117]. Here, we describe the identification of 47 host-derived proteins as a component of the *R. microplus* saliva content. The NSAF levels for these host-derived proteins, which include serum albumin, fibrinogen, serotransferrin, cathelicidin, immunoglobulin G chains, and hemoglobin, among others are shown in Additional file 2: Table S2. These findings indicate that the occurrence of host-derived proteins and/or their fragments in tick saliva may represent a genuine and widespread recycling system employed by ticks. In our previous research, when comparing the saliva of partially and fully engorged ticks, we detected a significant number of heme-binding proteins derived from the host, such as serum albumin, hemopexin, apolipoprotein, and peroxiredoxin. In that study, we proposed a mechanism wherein, towards the end of feeding, the tick replaces hemelipoproteins with host-derived heme-carrier proteins [9]. However, in the current study, we only detected serum albumin as a host-derived heme-binding protein. Despite this finding, the presence of host-derived proteins in tick saliva strongly suggests a collaborative system between tick and host during blood feeding. The presence of fragments of host proteins could also be a mechanism to increase the saliva osmolality and consequently control the water content in the saliva. Further investigation is required to elucidate the role of these host-derived proteins in the tick-host relationship [118].

## Conclusions

Hard ticks feed for prolonged periods, sometimes lasting several days or weeks, during which they must evade host responses to establish long-term parasitism. Proteome analysis has yielded significant insights into tick salivary proteins, revealing both the upregulation of various secreted proteins and the downregulation of others. This plasticity in salivary protein expression, often referred to as the 'sialome switch,' appears to be associated with the ticks' need to overcome various host responses to their feeding. Additionally, it may be linked to an immune evasion mechanism. The usual mechanisms that pathogens use to evade adaptive immunity are antigenic variation and immunomodulator proteins; both alter antigen recognition by host immune responses and allow the parasite to persist. Tick saliva collected at different times during the feeding process has many proteins related to immune evasion mechanisms. The findings presented in this study, along with the revealed sequences, enhance our comprehension of tick feeding biology and offer potential insights for discovering novel targets in the development of anti-tick strategies.

### Supplementary Information

**Additional file 1: Table S1.** Mass spectrometry data for tick proteins identified in the saliva of *Rhipicephalus microplus*. The summary (column B) outlines the validation criteria for protein identification within all groups of ticks. True proteins, identified in two out of the three technical replicates, are assigned a value of (1), while proteins detected in only one of the three runs were categorized as low confidence (0). This information is presented separately for groups Rm-1 (column C), Rm-2 (column S), Rm-3 (column AI), Rm-4 (column AY), Rm-5 (column BO), Rm-6 (column CE), Rm-7 (column CU), Rm-8 (column DK), and FEF (column EA). For each replicate, values representing unique peptides (UniquePeptideCount), peptide count (PeptideCount), spectral count (SpecCount), normalized spectral abundance factor (NSAF), and coverage percentage (Coverage) are provided.

**Additional file 2: Table S2.** Mass spectrometry data for bovine proteins identified in the saliva of *Rhipicephalus microplus*. The summary (column B) outlines the validation criteria for protein identification within all groups of ticks. True proteins, identified in two of the three technical replicates, are assigned a value of (1), while proteins detected in only one of the three runs were categorized as low confidence (0). This information is presented separately for groups Rm-1 (column C), Rm-2 (column S), Rm-3 (column AI), Rm-4 (column AY), Rm-5 (column BO), Rm-6 (column CE), Rm-7 (column CU), Rm-8 (column DK), and FEF (column EA). For each replicate, values representing unique peptides (UniquePeptideCount), peptide count (PeptideCount), spectral count (SpecCount), normalized spectral abundance factor (NSAF), and coverage percentage (Coverage) are provided.

**Additional file 3: Table S3.** A Windows-compatible hyperlinked Excel file that includes functional annotations, best reciprocal hits, and AlphaFold2 predictions for proteins identified in the proteome of *Rhipicephalus microplus* saliva. This file can be downloaded as a single.zip file from the following link: https://proj-bip-prod-publicread.s3.amazonaws.com/transcriptome/Rhip_microplus/Rm-saliva-2023/Table+S3.zip

**Additional file 4: Fig. S1.** Expression patterns of the proteases identified in the *Rhipicephalus microplus* saliva proteome throughout blood feeding. Dots represent the average of NSAF values of each functional group with error bars denoting the standard error.

**Additional file 5: Fig. S2.** Amino acid alignment (ClustalW) of proteins identified within the M12B, M13, and M17 families of metalloproteases identified in the *Rhipicephalus microplus* saliva proteome throughout blood feeding. The motif H-E-x(2)-H–x(2)-G-x(2)-H presented in metalloproteases is highlighted by a red box. The highly conserved residues are labeled in black, and the less conserved ones are in gray.

**Additional file 6: Fig. S3.** Amino acid alignment (ClustalW) of proteins identified within the S01 family of serine-proteases identified in the *Rhipicephalus microplus* saliva proteome throughout blood feeding and the trypsinogen anionic precursor from *Bos taurus* (AA38513.1). The catalytic triad comprising Asp, His, and Ser is highlighted by asterisks. The highly conserved residues are labeled in black, and the less conserved ones are in gray.

**Additional file 7: Fig. S4.** Amino acid alignment (ClustalW) of proteins identified within the C01 family of serine-proteases identified in the *Rhipicephalus microplus* saliva proteome throughout blood feeding and the cathepsin B from *Homo sapiens* (PDB 1GMY chain A). The catalytic dyad comprising Cys and His is highlighted by asterisks. The highly conserved residues are labeled in black, and the less conserved ones are in gray.

**Additional file 8: Fig. S5.** Expression patterns of the protease inhibitors identified in the *Rhipicephalus microplus* saliva proteome throughout blood feeding. Each data point represents the average NSAF value for each protease family, with error bars denoting the standard error.

**Additional file 9: Fig. S6.** Amino acid alignment (ClustalW) of proteins identified within the TIL family of protease inhibitors identified in the *Rhipicephalus microplus* saliva proteome throughout blood feeding and the chymotrypsin inhibitor from *Apis mellifera* (PDB 1CCV). The putative P1 residues are highlighted by asterisks. The highly conserved residues are labeled in black, and the less conserved ones are in gray.

**Additional file 10: Fig. S7.** Amino acid alignment (ClustalW) of proteins identified within the 8.9 kDa family of secreted conserved proteins identified in the *Rhipicephalus microplus* saliva proteome throughout blood feeding and the tick complement inhibitor CirpT1 from *Rhipicephalus pulchellus* (PDB 6RPT). The specific block of eight residues in the interaction interface between CirpT1 and C5 was identified [59] and residues are highlighted by asterisks. The highly conserved residues are labeled in black, and the less conserved ones are in gray.

**Additional file 11: Fig. S8.** Expression patterns of the secreted conserved proteins identified in the *Rhipicephalus microplus* saliva proteome throughout blood feeding. Each data point represents the average NSAF value for each protease family, with error bars denoting the standard error.

**Additional file 12: Fig. S9.** Amino acid alignment (ClustalW) of CirpT1 homologs identified in the *Rhipicephalus microplus* saliva proteome and in the saliva proteome of other tick species and the tick complement inhibitor CirpT1 from *Rhipicephalus pulchellus* (PDB 6RPT). The specific block of eight residues in the interaction interface between CirpT1 and C5 was identified [59] and residues are highlighted by asterisks. The highly conserved residues are labeled in black, and the less conserved ones are in gray.

## Data Availability

The proteomic data have been deposited in the ProteomeXchange Consortium database via the PRIDE partner repository. To facilitate the exploration of this dataset, we developed a R shiny application that can be accessed online (https://tickproject.shinyapps.io/RmSaliva/). Additionally, a Windows-compatible hyperlinked Excel file that includes functional annotations, best reciprocal hits, and AlphaFold2 predictions for proteins identified in this study can be downloaded as a single.zip file from the following link: https://proj-bip-prod-publicread.s3.amazonaws.com/transcriptome/Rhip_microplus/Rm-saliva-2023/Table+S3.zip
