# Supplementary material for: Changes in saliva protein profile throughout Rhipicephalus microplus blood feeding
Source: Parasit Vectors. 2024 Jan 27;17:36. doi: 10.1186/s13071-024-06136-5 (PMC10821567; doi:10.1186/s13071-024-06136-5)
Supplement: Supplementary file 3 — Additional file 3: Table S3. A Windows-compatible hyperlinked Excel file that includes functional annotations, best reciprocal hits, and AlphaFold2 predictions for proteins identified in the proteome of Rhipicephalus microplus saliva. This file can be downloaded as a single.zip file from the following link: https://proj-bip-prod-publicread.s3.amazonaws.com/transcriptome/Rhip_microplus/Rm-saliva-2023/Table+S3.zip [file 13071_2024_6136_MOESM3_ESM.pdf]

Additional file 3: Table S3. A Windows-compatible hyperlinked Excel file that includes functional annotations, best reciprocal hits, and AlphaFold2 predictions for proteins identified in the proteome of *Rhipicephalus microplus* saliva. This file can be downloaded as a single .zip file from the following link: https://proj-bip-prod-publicread.s3.amazonaws.com/transcriptome/Rhip_microplus/Rm-saliva-2023/Table+S3.zip
