# Supplementary material for: Changes in saliva protein profile throughout Rhipicephalus microplus blood feeding
Source: Parasit Vectors. 2024 Jan 27;17:36. doi: 10.1186/s13071-024-06136-5 (PMC10821567; doi:10.1186/s13071-024-06136-5)
Supplement: Supplementary file 4 — Additional file 4: Fig. S1. Expression patterns of the proteases identified in the Rhipicephalus microplus saliva proteome throughout blood feeding. Dots represent the average of NSAF values of each functional group with error bars denoting the standard error. [file 13071_2024_6136_MOESM4_ESM.pdf]

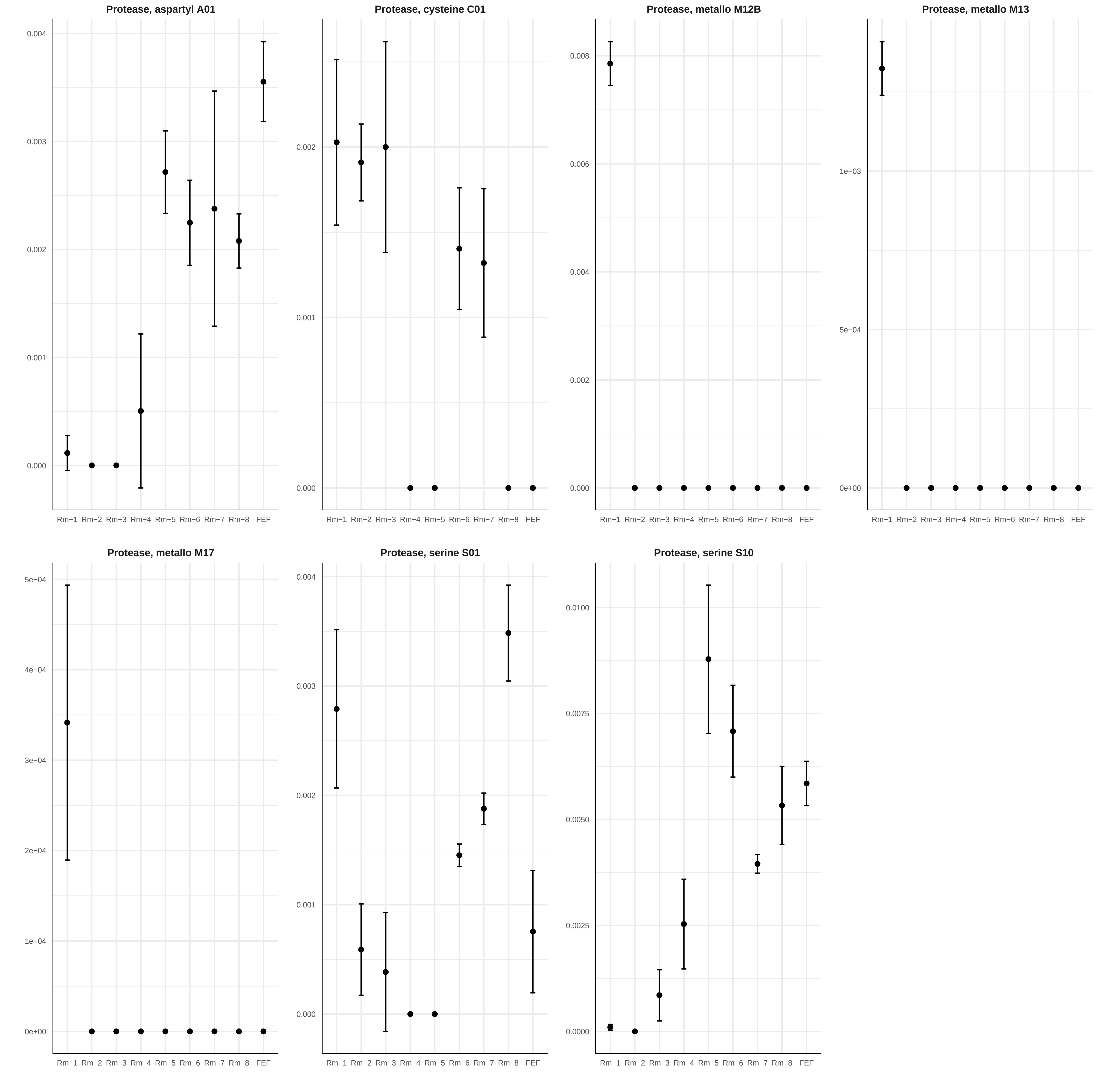
Protease, aspartyl A01
Protease, cysteine C01
Protease, metallo M12B
Protease, metallo M13
Protease, metallo M17
Protease, serine S01
Protease, serine S10
Rm−1 Rm−2 Rm−3 Rm−4 Rm−5 Rm−6 Rm−7 Rm−8 FEF
0.000 0.001 0.002 0.003 0.004
0.000 0.002 0.004 0.006 0.008
0e+00 5e−04 1e−03
0e+00 1e−04 2e−04 3e−04 4e−04 5e−04
0.0000 0.0025 0.0050 0.0075 0.0100
