# Supplementary material for: Changes in saliva protein profile throughout Rhipicephalus microplus blood feeding
Source: Parasit Vectors. 2024 Jan 27;17:36. doi: 10.1186/s13071-024-06136-5 (PMC10821567; doi:10.1186/s13071-024-06136-5)
Supplement: Supplementary file 5 — Additional file 5: Fig. S2. Amino acid alignment (ClustalW) of proteins identified within the M12B, M13, and M17 families of metalloproteases identified in the Rhipicephalus microplus saliva proteome throughout blood feeding. The motif H-E-x(2)-H–x(2)-G-x(2)-H presented in metalloproteases is highlighted by a red box. The highly conserved residues are labeled in black, and the less conserved ones are in gray. [file 13071_2024_6136_MOESM5_ESM.pdf]

```
Rm-149460 : -------------------------------------------------- :   -
Rm-1513   : -------------------------------------MIPLLRSLSVACS :  13
Rm-156308 : -------------------------------------------------- :   -
Rm-18262  : -------------------------------------------------- :   -
Rm-24243  : -------------------------------------------------- :   -
Rm-2425   : -------------------------------------------------- :   -
Rm-47575  : -------------------------------------------------- :   -
Rm-47576  : ---------------------TKVLKINEDITLNLEPSSILHENFFVRSY :  29
Rm-5080   : AKLPKYRAITYPQVFDGRDENTKVVKINDDISLNLEPISVLHEDFFIRTY :  50
Rm-63410  : -------------------------------------------------- :   -
Rm-7655   : -------------------------------------------------- :   -
Rm-78386  : --AMAEQHVYPRILYERSTSGNLVLKLNEKITLNLEKSTVLADNLLFVTT :  48

Rm-149460 : ---------------------------SVHVRRRGGGVEVEGVVGPRHRI :  23
Rm-1513   : KSNPYVLSPVLSAHRRSLAMAAKRQAVLLGVYQDKDKDSEGDFVLTPSAK :  63
Rm-156308 : -------------------------------------------------- :   -
Rm-18262  : -------------------------------------------------- :   -
Rm-24243  : -------------------------------------------------- :   -
Rm-2425   : ----MADTAQLLFPVTVESSESAEFDCVVIVGTGLDATNLQGGILDKYRE :  46
Rm-47575  : -------------------------------------------------- :   -
Rm-47576  : KKGIPEHQYYDIRDLQKNFYHDKKQFAALMLYEEEGTLRVEGVVGPNLKI :  79
Rm-5080   : RNGIAEHHYHNVRELQKDLYHDKKRYAAVMLSEEDGTVRVEGVVGTNLKI : 100
Rm-63410  : -------------------------------------------------- :   -
Rm-7655   : ----------------------------------AKGVSMRGLVGPHHRI :  16
Rm-78386  : NKDLDEVEMIDTSHIQRTLFHDTHYQSSVIVRQRDDNVEVEGIINDNLRI :  98

Rm-149460 : EPVHTMERSEEGLIAHMIHEIE-MKDMHDIVMAPIGKGAPTVSERNYG-- :  70
Rm-1513   : QFAASSKLTDLLELTKGFFKKGETRIFYGLDEKYPITSVVHLGPRQAEVR : 113
Rm-156308 : -------------------------------------------------- :   -
Rm-18262  : -----GEEMQNEEQQEEAIRLG---------------------------- :  17
Rm-24243  : ------QEMQAEEHEEEVIKLG---------------------------- :  16
Rm-2425   : PLVAYKQVDKNAEDEVFVLNLPEKFSIRRLVYSPTGPLNRDFDDVRAFAD :  96
Rm-47575  : -------------------------------------------------- :   -
Rm-47576  : RPIESTERSEHGQQAHLVDTIE--DKDSVEVYGEFARNKVDISERAKSSG : 127
Rm-5080   : RPIETAERSDDGRLAHLVENIE--EDDSFEVYGKVVEDSIGISERAARSK : 148
Rm-63410  : -----GRGSQQGQILHSLFEVP-VTEEFSEMEEEIPRL-RERSNHNTSSS :  43
Rm-7655   : QPMPVSERSEEDTIPHMIYKID-AHNMLDKTLKTTERSRAKISERAYG-- :  63
Rm-78386  : KPLPEGQRSTNGQMLHTIFQVPTIKENFTETKKHFLGLERSKRNDIKGYR : 148

Rm-149460 : -------STQNVPPSVTIELFVVSDRPHHQHFQTTIQLIQYLCVMVNSMN : 113
Rm-1513   : ELEECDEAAESVRIAVAAGVRSLRSVGATAIDVDPCANAEAAAEGCTLAL : 163
Rm-156308 : -------------------------------------------------- :   -
Rm-18262  : -----------------IYFLYDQAFWNGALFVKNNSYNAYFAALTRAAQ :  50
Rm-24243  : -----------------LQFVYDEAFLNRSLFILKGSYDAYFSALTRAAE :  49
Rm-2425   : AACKGVKRATAAGSTKPLVVLPAKSGSFKSYDVVTVLGTLHGLYVPLEIR : 146
Rm-47575  : ----------------------LCDSVFQQKFKKREHIIPYLMTTIQVVN :  28
Rm-47576  : ---TGFDPTKYAVPIIYPELYVLCDSVFQQKFKKREHIIPYLMTTIQVVN : 174
Rm-5080   : ---SGFDRTKYNVYEMFPEVFVVCDSWFQAEFVKSMNVTVYMIITFQVVS : 195
Rm-63410  : DWANAPQSRSAGLQRFPVEVHIVSDKAHQQHFKDNTKLISYLAVMMNAAK :  93
Rm-7655   : -------RPTPVPDTIHVEVFIVTGKRHIAYYKETTYLIWYACVMVNFAN : 106
Rm-78386  : EDLSFLQARASRVDKFPVELHIVSDKEHQRSYRKNEELISYLAVMMNAVC : 198
```

```
Rm-149460 : LRYADTESPRVTFLLIGVQKDEHSP---YRNGNDK-YLESSTSLDRFRSY : 159
Rm-1513   : HVFEELKKKESRKLPVAVSPLNEQELDKWTRGLEKSAAQNLARRLSEMPA : 213
Rm-156308 : -------------------------------------------------K :   1
Rm-18262  : EFFKLHTDPKILLTLVGSSKLQDN-IVNNTITKGNTLNASETLEKLGAIL :  99
Rm-24243  : EFFKLHDDPKIKLTLVGSSKLQEEDIVTNTTTTDHKLNASETLDNLRTLL :  99
Rm-2425   : EDVPSRKQKVTRLGFANFPATVPNGDKSFKLALAIERARIVCRDIGGSDP : 196
Rm-47575  : IRYGTLSGPQVRIVLRGIELTDAKQEGRYYVYDGYG-IDAYASLQKLVTF :  77
Rm-47576  : IRYGTLSGPQVRIVLRGIELTDATQEGRYYVYDGYG-IDAYASLQKLVTF : 223
Rm-5080   : IRYSALRNPKVYPVLRGIELSTLQQEKKYYVYLNAASIDAYKSLLKLVTF : 245
Rm-63410  : LRYLDTTNPKIEFLLVGVTRVTDYE---FARIDGR-YIDAGEMIDGLKLY : 139
Rm-7655   : LRLEAITNPKVKLVLVGLEKDEKEP---YAVVTDDGYLFDELTIKEFQRY : 153
Rm-78386  : LRYLDMMNPKITFLLVGVTRAKDHD---FGRNNGG-EIDAAEMLRGLGQY : 244

Rm-149460 : AYGKRHQFGNPDVTFLVT-GYDVYSTGNGGSKSTSVLGIGYVGGLCT--- : 205
Rm-1513   : NMMTPTRFAQEAAEALEKKGVKVIARDRKWIEEQKMGSFLSVTQGSDQPP : 263
Rm-156308 : SIDNKRTYNVCETEACIR-RAKLIT-----------ESLNTSADPCT--- :  36
Rm-18262  : TWHNNTLYAGADVIFLAT-GLKLHITESW--RTGEWYGLSYRRSICFG-- : 144
Rm-24243  : TWN-DTLNPSVDVVFLVT-GMELVITESR--MTGECHGLAYPRSICFG-- : 143
Rm-2425   : ERMAAPRVAEYVQQVFKNSPVNVEILADDGRILKEYPLLAAVNRATKNVS : 246
Rm-47575  : AAAENQTYKTFDMMYFVT-GYDMIAVYTN-DRMSALEGYAFVGSACT--- : 122
Rm-47576  : AAAENQTYKTFDMMYFVT-GYDMIAVYTN-DRMSALEGYAFVGSACT--- : 268
Rm-5080   : VTERNDTYQTFDMVYFVT-RYDMVAVYDDGSRQNSLQGYAFVGSACS--- : 291
Rm-63410  : KSQGR-IHGHHDVVYLIS-GYDISKWLAKGKRYNGIRGRAKVGTVCT--- : 184
Rm-7655   : AVKRKDDFGRPDTVFLLS-GYDVITVHEG-RLTEAGLGIGYLSGICT--- : 198
Rm-78386  : RNQGR-IPGNFDVVYLLT-ALDMIR-FSKGKKIKGIAGRAKMSTACT--- : 288

                                                HExxHxxGxxH
Rm-149460 : ---------EHFVALGEDSAGLYTGMHTLTHECGHVLGAAHDESRPT-TW : 245
Rm-1513   : ---VFLEMHYEGPGAVAGGPLVFVGKGVTFDSGGISLKPSANMDRMKADM : 310
Rm-156308 : ---------------------DFY---------SYACGG----------- :  45
Rm-18262  : ---------NSTVGIIYDDGANFYGVRLMALQVALLLGAWKDNGRWG--- : 182
Rm-24243  : ---------NATVGIIHDDGATFNGVRLAALQIAFLLGAKKDNGKWG--- : 181
Rm-2425   : RHHCRMIHLTYTPEGPIKKTLFLVGKGVTYDTGGADVKAGGHMAGMHRDK : 296
Rm-47575  : ---------THRQLLGEDTAYTFKGIRIVTHEIGHALGCSHDGTSAPGIV : 163
Rm-47576  : ---------THRQLLGEDTAYTFRGIRIVTHEIGHALGCSHDGTSAPGIV : 309
Rm-5080   : ---------KNREQLGEDTAYSYRGIRTMTHELAHALGCSHDGTAAPGIV : 332
Rm-63410  : ---------ELGISEGEDRPHGYLGVNTIAHELGHTLGAVHDHTP----- : 220
Rm-7655   : ---------NAYVALGEDKPGLFTGAHTFTHELAHLLGASHDGEDAK-SD : 238
Rm-78386  : ---------ERGLGEGEDTPHAYSGVNTFAHELAHTLGSDHDETP----- : 324

Rm-149460 : IQGDPGSMKCLWKEGY--IMSYVDGGVKHHH-FSPCSLAQIQNLVKLRGP : 292
Rm-1513   : TGAACVVATFAAVAALK-LPVKMVGLAPLCENLPSGRATKPGDVFTAMNG : 359
Rm-156308 : -----------WMAKHT-IPETKSSTGGFYL-LADQLKETLRDILGNV-- :  80
Rm-18262  : ---------ECPKNEEEE-YLTSNPRGGRIPY-LSECSRESVRSFYYRVKS : 222
Rm-24243  : ---------ECPKNEEQ--YLTSNCSGGHIPR-LSDCSKSSVRDFYYRAKD : 220
Rm-2425   : CGAAAVAGFFKVLSELRPKGVKVYGAMAMVRNSIGSECYVSDEIITSRAG : 346
Rm-47575  : KAFVPNSLHCPWGDGY--IMSYEQHDSRSMR-FSSCCRYDISQMSWSREA : 210
Rm-47576  : KAFVPNSLHCPWGDGY--IMSYEQHDSRSMW-FSSCCRYDISQMSWSREA : 356
Rm-5080   : TAFTPDSLQCPWEHGY--IMSYYEIDIRSMQ-FSRCCRYDIQRMSWAYEG : 379
Rm-63410  : ---------ECPWKDGY--LMSYEDGGLKKFR-LSQCSERSIRNYVRTLSN : 259
Rm-7655   : VPGHPSATTCPWKQGF--IMSYVNNGPSHHR-FSHCSVTQIRHVLTYRGP : 285
Rm-78386  : ---------QCPWEDGY--LMSYVDGGLRKYK-LSQCSQNSIRQYVGRLSD : 363
```

```
Rm-149460 : SCWQLGNTGHTNDG-----VYPGMEVAPNAY----CRHAFPDKENVTADM : 333
Rm-1513   : TTIQVDNTDAEGRLILADALCYADTFNPKVV----VDIATLTGAMVVALG : 405
Rm-156308 : --------------------------------------------------  :   -
Rm-18262  : N-RDICWNDKPKPALDEEIGFPKD-FYKLFD----CDHCHVAEHFRNKTT : 266
Rm-24243  : GGYTLCWNDTPEPALPNNTDFPVD-FYRQFD----CDQCHVAEHLKNNTG : 265
Rm-2425   : VRVRIGNTDAEGRMAMADALCECKEKALNEVNPRIFTIATLTGHACIAVG : 396
Rm-47575  : NCLHVNDSMKYPLNWLIKYKLPGDFLSLNRQ----CEIAYPNLWRTYYVQ : 256
Rm-47576  : S------------------------------------------------- : 357
Rm-5080   : GCLHKNNSNRFPLNWINEYYLPGENLSLNRQ----CEMKYPQLHGTYYLE : 425
Rm-63410  : DCIKVLNSQNYMRNQR---KFPGETIRKKYY----CRKKVGNTK-ESKSV : 301
Rm-7655   : TCWAFNGSGYSMRD-----TYPGMVVSFEQF----CMGLLNNKANTTIRD : 326
Rm-78386  : KCISVLSRQNYMKGRN---KYPGQTIRKNYY----CRRLMQKRA-KGQKI : 405

Rm-149460 : ESDSMKKCMVKCQYPEYRRVCYGRCFIYVTTYSLELHALDYMPCGGN--- : 380
Rm-1513   : AGATGVFCTSNALWNLLHEAGSVTGDRVWRMPLFDLYHKQMTKSTVA--- : 452
Rm-156308 : -------------------------------------------------- :   -
Rm-18262  : QPINCSLSTINRNINNWPSTKESDWARKARKRYERWYRKHTTTVSPY--- : 313
Rm-24243  : KPINCSMSTINRDINNWPSTTESSWHRAARKRYRQWYWKRTTTVSPY--- : 312
Rm-2425   : DGYSIIMDNGPARSNAVSATVQAAGDEIGDMFEVSTVRREDFNFVCGPSE : 446
Rm-47575  : KTH-KWYCKGYCFVPGHQFRAADHY--------------WDFLFVDG--- : 288
Rm-47576  : -------------------------------------------------- :   -
Rm-5080   : KVIDKWYCQGYCYVPEVPRESSAGQ--------------WDFLFMDG--- : 458
Rm-63410  : YVEKDNGCFLQCCIKHPSHTWCGKY-----------KMLDGMTCDPG--- : 337
Rm-7655   : VTVDIATCKVTCTYTRLTYRLYDDS-VYKSTIQTKDDALDYMPCGDY--- : 372
Rm-78386  : IVQKAADCDIKCCRMGDGYMSCATY-----------KMLDGMECAQG--- : 441

Rm-149460 : -----QVCIQGVCGEG--KIVMPSQKPTTVTVTADTEATTTTTTT---TT : 420
Rm-1513   : -----DINNISKQAGAGGSCVAAAFLHEFVKCPQWAHLDIAGVMENKDEV : 497
Rm-156308 : -------------------------------------------------- :   -
Rm-18262  : -----HACTQSCCRFMRYTDRYGGWWDCWDTRAADGTVCDSTRVCLDGEC : 358
Rm-24243  : -----KRCTQSCCRFLRHGPDRGGWWDCWYTPAADGTVCDSTRVCLDKAC : 357
Rm-2425   : YEDLIQCNNEPSSRTPRGHQMPAAFLIKASGLDKHGLDSERPLCYSHIDI : 496
Rm-47575  : -----TVCMNSIAS-RHHNWVTPLFYSQNTT------------------- : 313
Rm-47576  : -------------------------------------------------- :   -
Rm-5080   : -----TTCHDGYXXXHLYKRRMPGGPEKNRTT------------------ : 485
Rm-63410  : -----KTCRRGVCAKH---------------------------------- : 348
Rm-7655   : -----KVCIQGYCVPKPTDVTDASKVPDNVTPTTTTTTTTQGTTEESGTD : 417
Rm-78386  : -----KTCRRGVCGYH---------------------------------- : 452

Rm-149460 : TPTTASTECRCDCSTTASSTQTRDQGERTS----PRTRRPERLGNRWWYY : 466
Rm-1513   : PYLGSGMAGRPVRTLVEFVERAAKLEKL---------------------- : 525
Rm-156308 : -------------------------------------------------- :   -
Rm-18262  : A------------------------------------------------- : 359
Rm-24243  : G------------------------------------------------- : 358
Rm-2425   : AASSGPFPGVPTGAPIAALTQAFLVE------------------------ : 522
Rm-47575  : -------------------------------------------------- :   -
Rm-47576  : -------------------------------------------------- :   -
Rm-5080   : -------------------------------------------------- :   -
Rm-63410  : -------------------------------------------------- :   -
Rm-7655   : SSSGTSTECKCDCLSTTGTTPTGTTSTRTTTTLMPSSNRPRFFKHRPWSR : 467
Rm-78386  : -------------------------------------------------- :   -
```

Rm-149460	GK	468
Rm-1513	--	-
Rm-156308	--	-
Rm-18262	--	-
Rm-24243	--	-
Rm-2425	--	-
Rm-47575	--	-
Rm-47576	--	-
Rm-5080	--	-
Rm-63410	--	-
Rm-7655	G-	468
Rm-78386	--	-
