# Supplementary material for: Changes in saliva protein profile throughout Rhipicephalus microplus blood feeding
Source: Parasit Vectors. 2024 Jan 27;17:36. doi: 10.1186/s13071-024-06136-5 (PMC10821567; doi:10.1186/s13071-024-06136-5)
Supplement: Supplementary file 6 — Additional file 6: Fig. S3. Amino acid alignment (ClustalW) of proteins identified within the S01 family of serine-proteases identified in the Rhipicephalus microplus saliva proteome throughout blood feeding and the trypsinogen anionic precursor from Bos taurus (AA38513.1). The catalytic triad comprising Asp, His, and Ser is highlighted by asterisks. The highly conserved residues are labeled in black, and the less conserved ones are in gray. [file 13071_2024_6136_MOESM6_ESM.pdf]

```
Rm-2605     : YFGGDPKTGPEGGVPLGECVCVPYYQCKEGKVSEDGGGLLDARRKLPPKE :  50
Rm-77083    : -------------------------------------------------- :   -
Rm-163513   : -------------------------------------------------- :   -
AA38513.1   : -------------------------------------------------- :   -

Rm-2605     : EIPLDGLGDQNQQCPGVDQVCCAEPSAVTETPYVASCGIRNDNGINSRIL : 100
Rm-77083    : -------------------------------------------------- :   -
Rm-163513   : EKTMDSIMEKLLIGLSLLFVTLERMSAQKETLNPKGCG------VSSYKT :  44
AA38513.1   : -------------------------------------------------- :   -

                                                         *
Rm-2605     : TKDGKGEAEFGEWPWQAAVLKYESEILKFECGGTLIASRYVLTVAHCVAR : 150
Rm-77083    : --VGGRVADVGAWPWMAAIYLKTEAQPKVGCGGALVTDRHVLTAAHCVSV :  48
Rm-163513   : MIVNGTEVKETQYPWAVFLATQFPSG-QYACGGTIITKRHVLSAAHCFFV :  93
AA38513.1   : -IVGGYTCAENSVPYQVSLNAG-----YHFCGGSLINDQWVVSAAHCYQY :  44

Rm-2605     : FNGADR---VPLKVRLGEWDTQSMKEFYPHEDYDVGNIYIHQYFRNNSLW : 197
Rm-77083    : GARARQLPARVLTVRVGDHDLNSSDDNTTPMDVEVADVIRHPRYDRRTYA :  98
Rm-163513   : NNEYAQ----KVTVSYGSVDRHSGK------KVDASKVLIHKHFDNLTAS : 133
AA38513.1   : -----------HIQVRLGEYNIDVLEGGEQ--FIDASKIIRHPKYSSWTLD :  82

               *
Rm-2605     : NDIALLELTRPVTFAPHISPICLP---KLEDVFEGTSCVVTGWGKDAYRT : 244
Rm-77083    : NDIALLVLRKPVTWGRYVMPVCLPYGPLSSNTLEGHNAFIVGWGATQFNG : 148
Rm-163513   : NDIALLEVKYPFQFSKDVAPICLP---TTPVKLVNKDAVVAGWG-SLYLG : 179
AA38513.1   : NDILLIKLSTPAVINARVSTLLLP----SACASAGTECLISGWGNTLSSG : 128

Rm-2605     : GKFANIMKEVTVPVIDNPMCQNLLRQTRLGRYFRLHEGFICAGTEDG-VD : 293
Rm-77083    : AG-SSVLRQAQIPVWAEAECKKSYAQH-----LPISKAQLCAGDAGAEMD : 192
Rm-163513   : GQGVDFLRHTTVTFLPDQICSVIFMGR------RYSSVLQCCAHKRG-KG : 222
AA38513.1   : VNYPDLLQCLVAPLLSHADCEASYPG-------QITNNMICAGFLEGGKD : 171

                   *
Rm-2605     : SCKGDGGGPLSCYTPDGRYHLAGLVAWGI-DCGTPDVPGVYVRVAKYLDW : 342
Rm-77083    : SCQGDSGGPLLLPHEG-RYYVVGIVSSGK-DCATPNFPGIYTRVSSYLDW : 240
Rm-163513   : ACKGDSGGPVMLRSATDRFQQVGIVSYLIGTCGGDFNPQVYTRINAYIDW : 272
AA38513.1   : SCQGDSGGPVACNGQ-----LQGIVSWGY-GCAQKGKPGVYTKVCNYVDW : 215

Rm-2605     : ISEVTRLPISEFYPK---------------- : 357
Rm-77083    : LRDQLGVGQQ--------------------- : 250
Rm-163513   : LTQAVSSSASYKLLGSPKPEMLKRISISWG : 302
AA38513.1   : IQETIAANS---------------------- : 224
```
