# Supplementary material for: Changes in saliva protein profile throughout Rhipicephalus microplus blood feeding
Source: Parasit Vectors. 2024 Jan 27;17:36. doi: 10.1186/s13071-024-06136-5 (PMC10821567; doi:10.1186/s13071-024-06136-5)
Supplement: Supplementary file 7 — Additional file 7: Fig. S4. Amino acid alignment (ClustalW) of proteins identified within the C01 family of serine-proteases identified in the Rhipicephalus microplus saliva proteome throughout blood feeding and the cathepsin B from Homo sapiens (PDB 1GMY chain A). The catalytic dyad comprising Cys and His is highlighted by asterisks. The highly conserved residues are labeled in black, and the less conserved ones are in gray. [file 13071_2024_6136_MOESM7_ESM.pdf]

```
Rm-16958    : --------VSEDAWRYMIPGQTDAFSDKMIQYINYLNTTWKAGRNPGFED :  42
Rm-5043     : LTHQELIGAEWSAFKALHGKDYESDTEEYYRLKIYMENRLKIARHNEKYA :  50
1GMY        : -------------------------------------------------- :   -

Rm-16958    : PAYVRSLLGV------------HPENQRYRLPERRLDLSSLGPLPENFDS :  80
Rm-5043     : KSQVSYKLAMNEFGDLLHHEFVSTRNGFQRNYRESPREGSFFVEPEGFEE : 100
1GMY        : ------------------------------------------KLPASFDA :   8

                                        *
Rm-16958    : REN-----WPECTTIGEIRDQGSCGSCWAFGAVEAMSDRTCIHSPPGGPK : 125
Rm-5043     : LHLPKAVDWRKKGAVTPVKNQGQCGSCWAFSTTGALEGQHFRKTR----- : 145
1GMY        : REQ-----WPQCPTIKEIRDQGSCGSCWAFGAVEAISDRICIHTN---AH :  50

Rm-16958    : RVVHLSADDLLSCC-KFCGDGCDGGFPGSAWNFWVK-KGIVTGGNYDSDD : 173
Rm-5043     : KLVSLSEQNLVDCSRSFGNNGCEGGLMDNAFKYIKANKGIDTEDTY---- : 191
1GMY        : VSVEVSAEDLLTCCGSMCGDGCNGGYPAEAWNFWTR-KGLVSGGLYESHV :  99

Rm-16958    : GCMPYPIKACDHHINGTLGPCDKKIPPTPRCVHMCRKGYDVHYDDDKHYG : 223
Rm-5043     : ---PYNGTDGVCHFNKSD-------------VGATDSGFVDIPEGD---- : 221
1GMY        : GCRPYSIPPCEHHVNGSRPPCTGEGD-TPKCSKICEPGYSPTYKQDKHYG : 148

Rm-16958    : KSGYSVPSTEEQIQAEIMTNGPVEADFTVYAD-FVHYKSGVYQR-HTDEA : 271
Rm-5043     : ---------ENKLKKAVAMVGPVSVAIDASHESFQFYSEGVYDEPECDSE : 262
1GMY        : YNSYSVSNSEKDIMAEIYKNGPVEGAFSVYSD-FLLYKSGVYQH-VTGEM : 196

                 *
Rm-16958    : LGGHAIRLLGWGVENGVPYWLAANSWNTEWGDKGFFKILRGSD-ECGIED : 320
Rm-5043     : QLDHGVLVVGYGTKDGQDYWLVKNSWGTSWGDGGYIYMSRNKENQCGIAT : 312
1GMY        : MGGHAIRILGWGVENGTPYWLVANSWNTDWGDNGFFKILRGQD-HCGIES : 245

Rm-16958    : DVVAGLPRY--------------- : 329
Rm-5043     : SASYPCRSSACSILLCRIHSPFRV : 336
1GMY        : EVVAGIPRTDQYWEKI-------- : 261
```
