# Supplementary figures and images for: Changes in saliva protein profile throughout Rhipicephalus microplus blood feeding
Source: Parasit Vectors. 2024 Jan 27;17:36. doi: 10.1186/s13071-024-06136-5 (PMC10821567; doi:10.1186/s13071-024-06136-5)

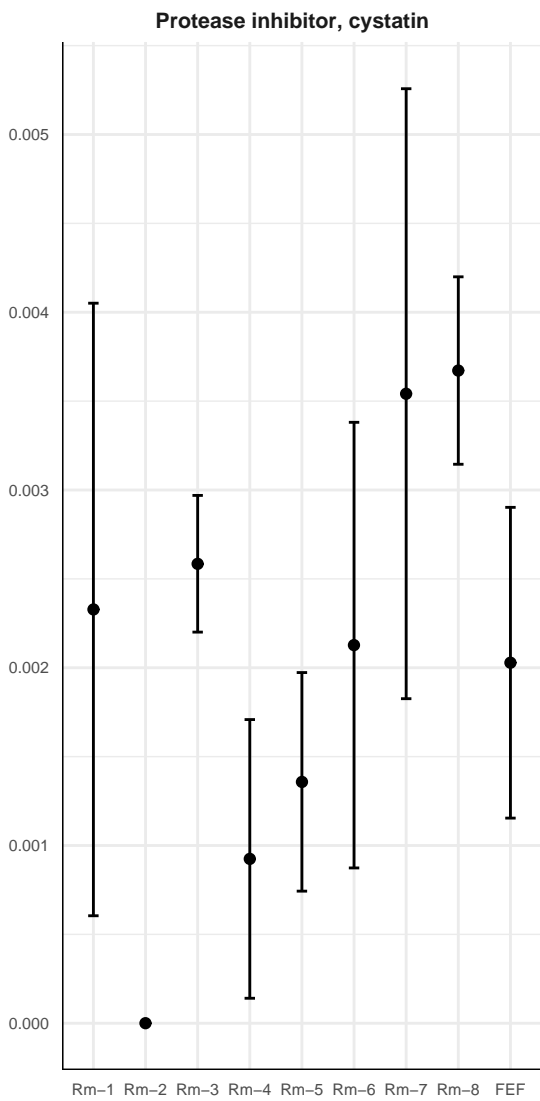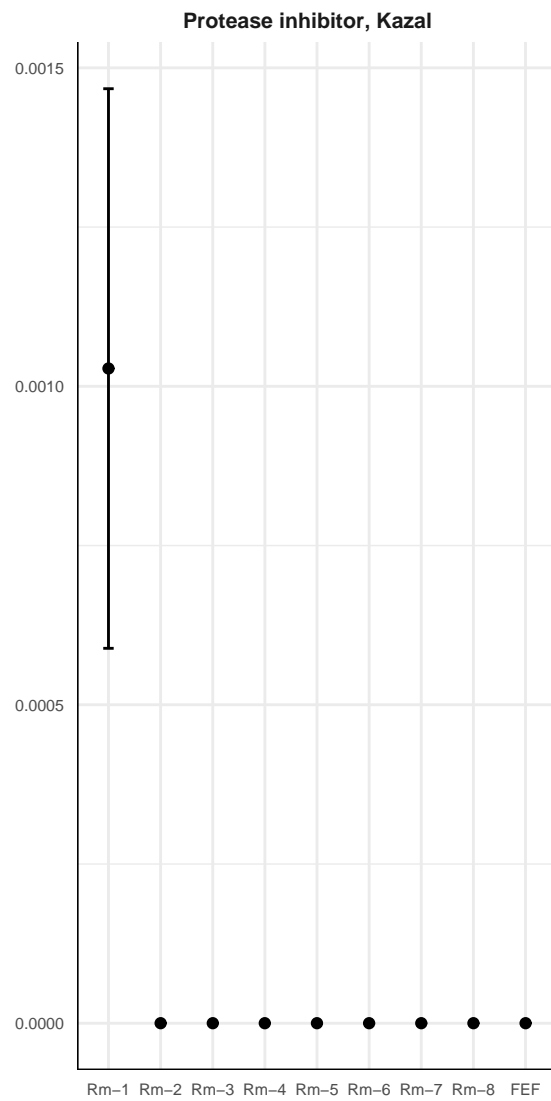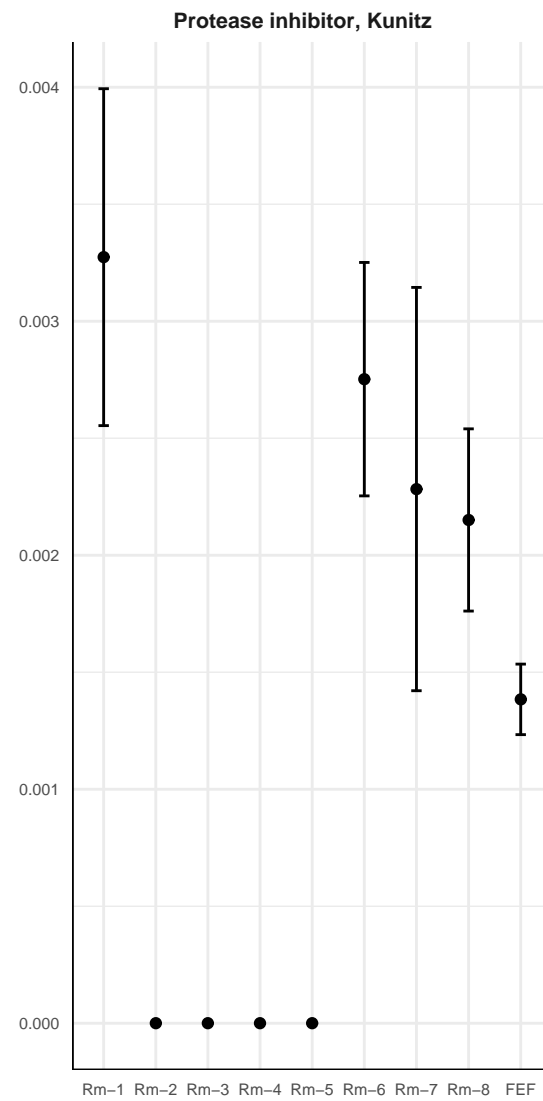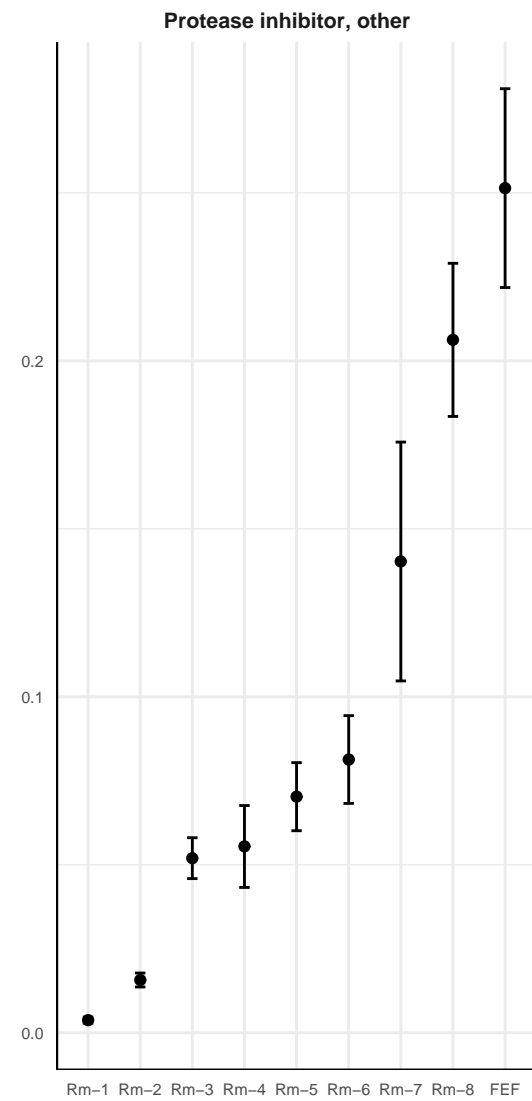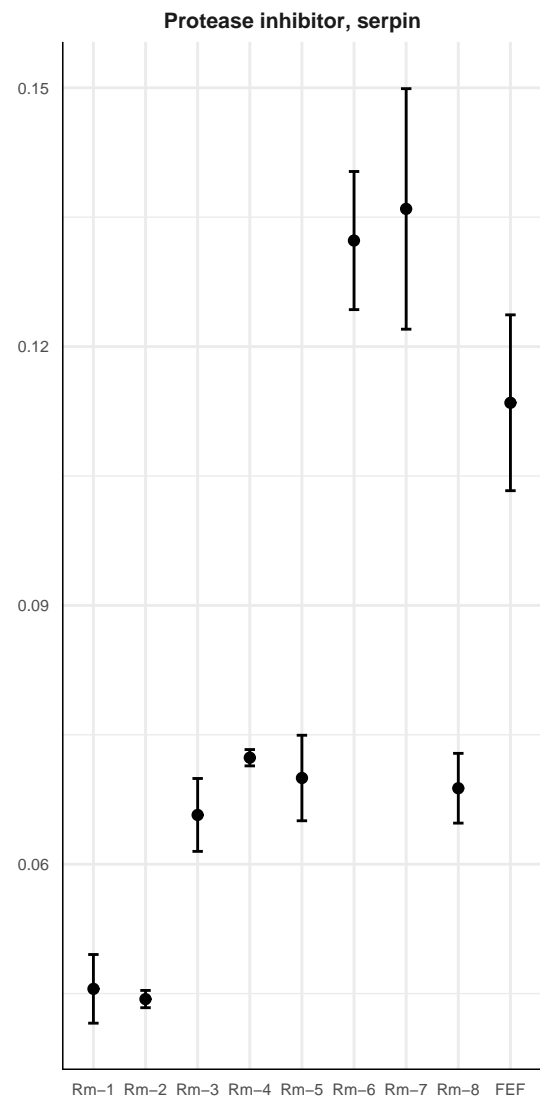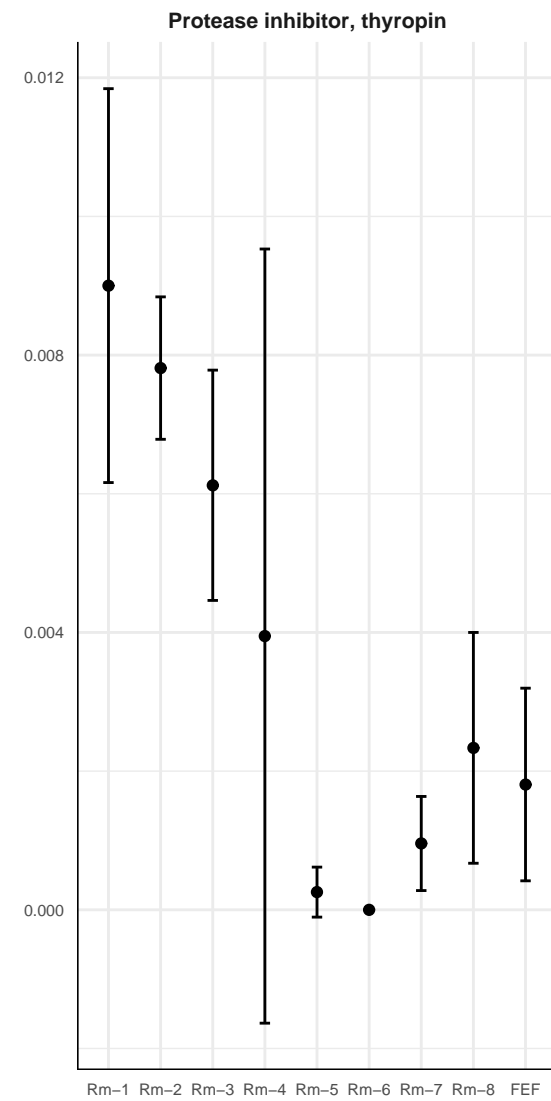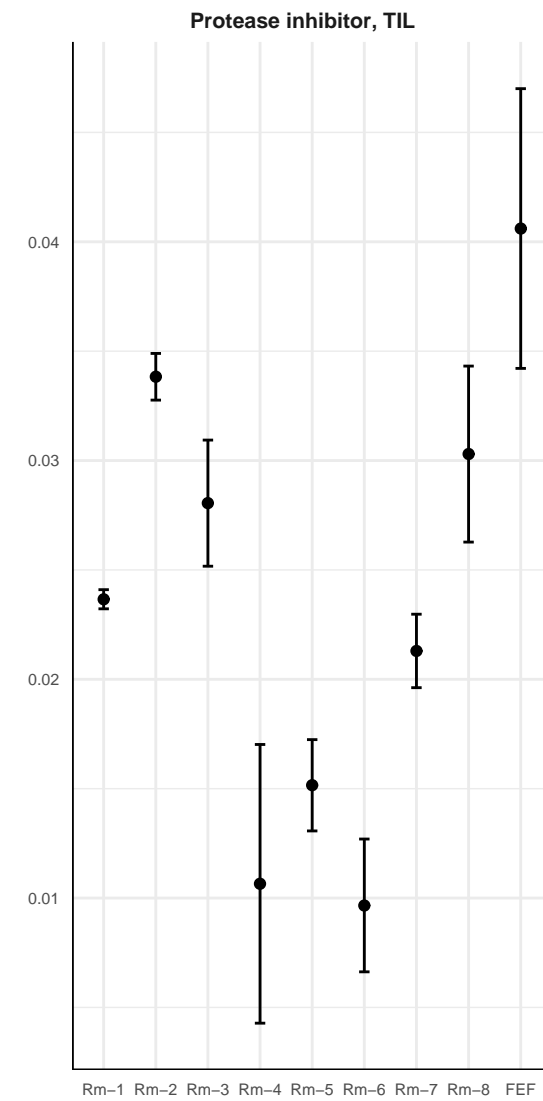

Supplement: Supplementary file 8 — Additional file 8: Fig. S5. Expression patterns of the protease inhibitors identified in the Rhipicephalus microplus saliva proteome throughout blood feeding. Each data point represents the average NSAF value for each protease family, with error bars denoting the standard error. [file 13071_2024_6136_MOESM8_ESM.pdf]

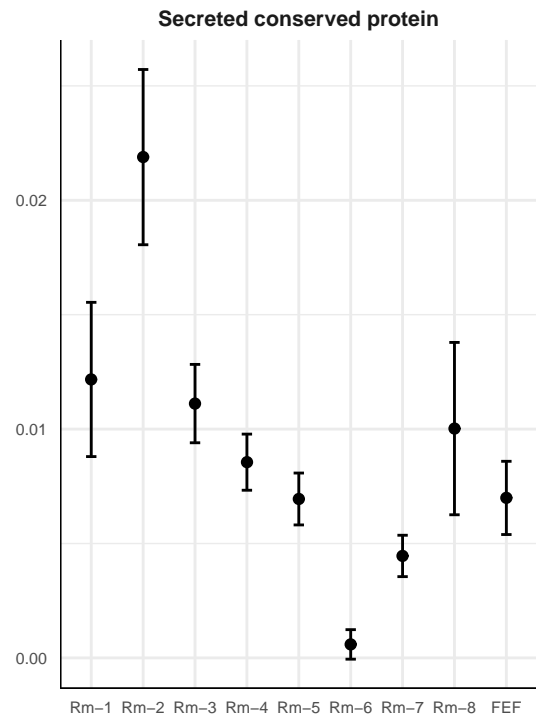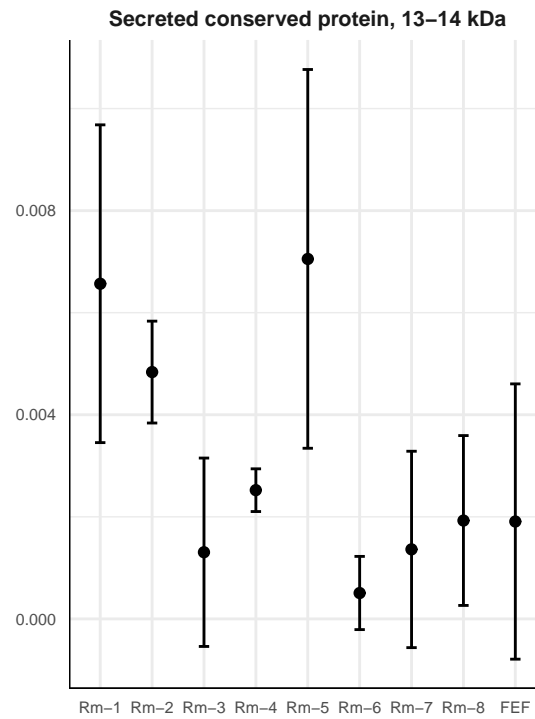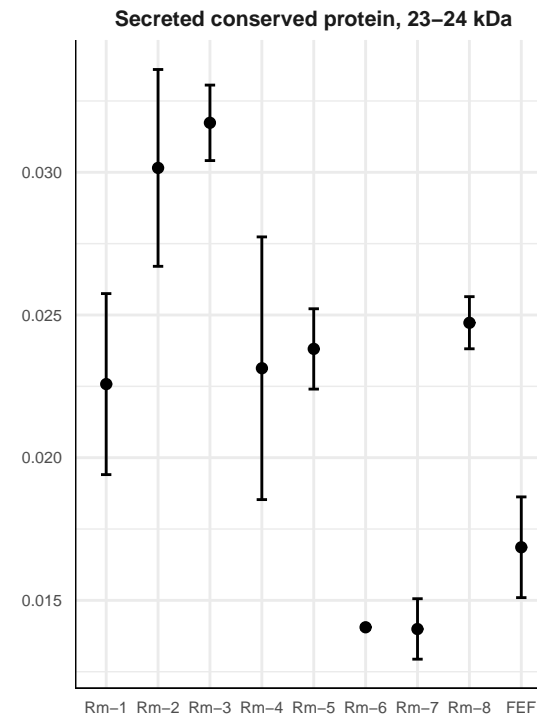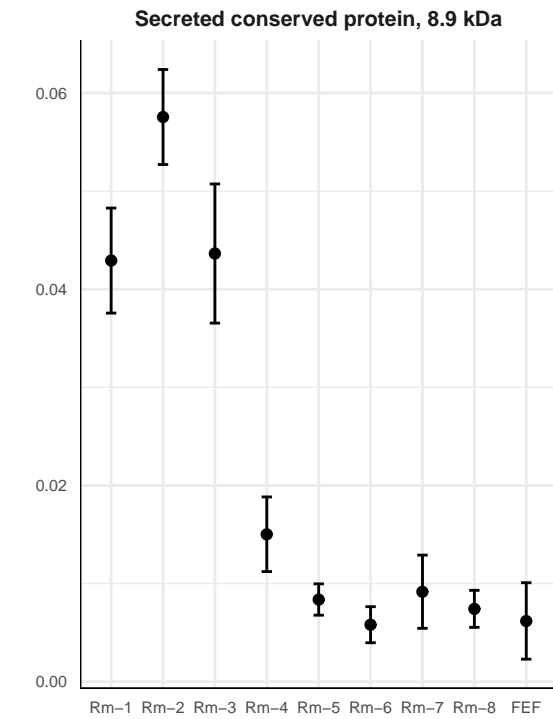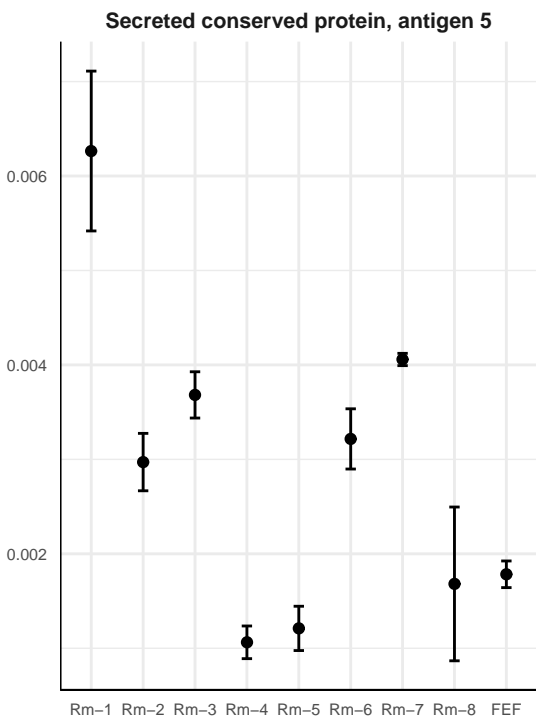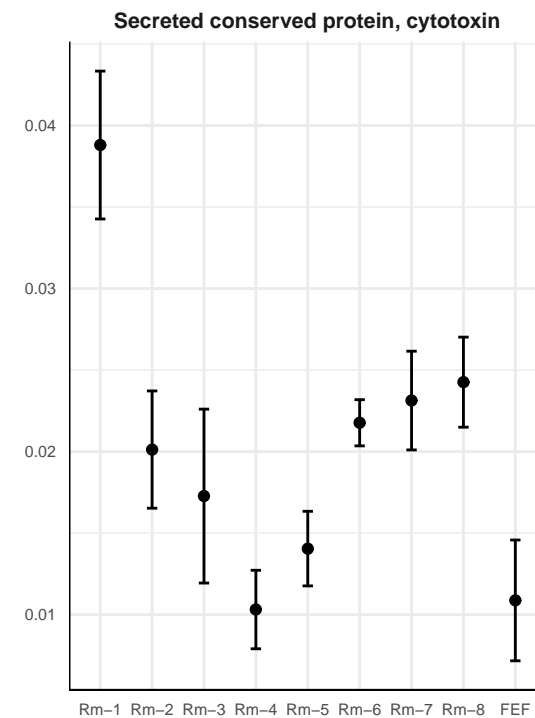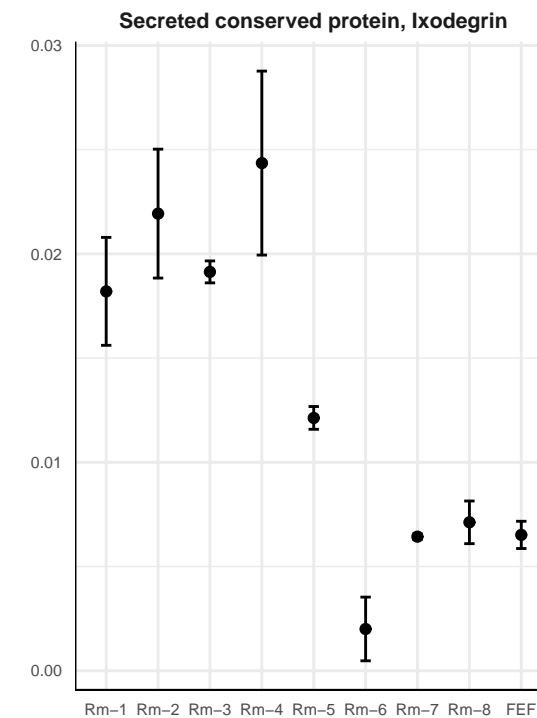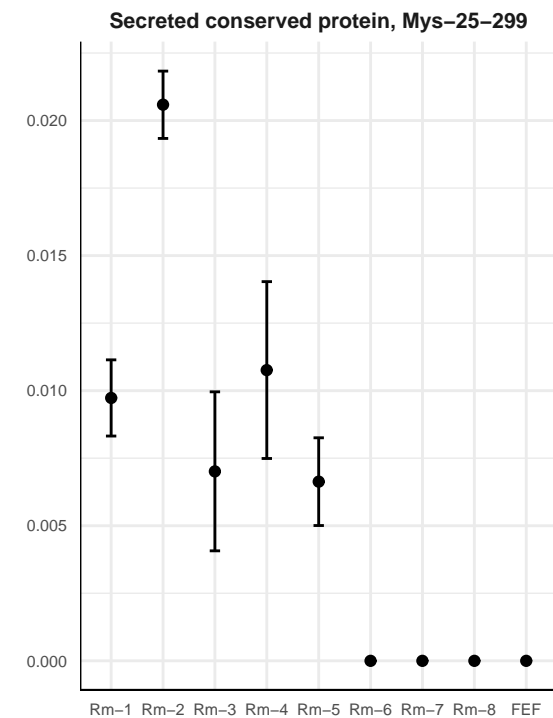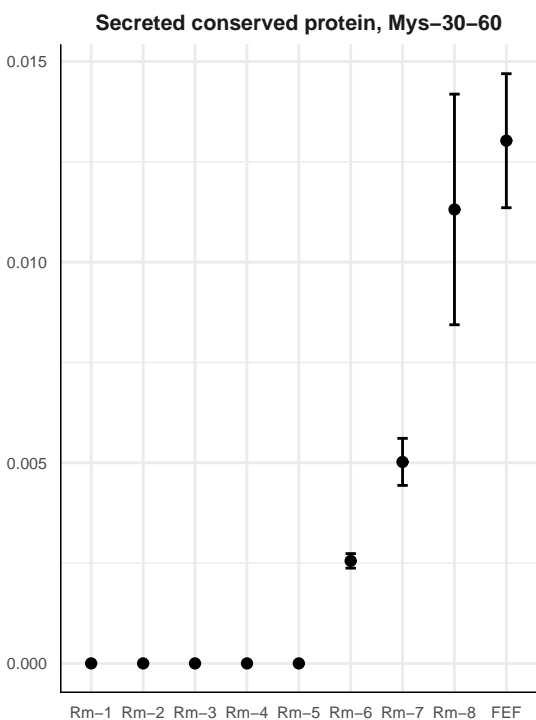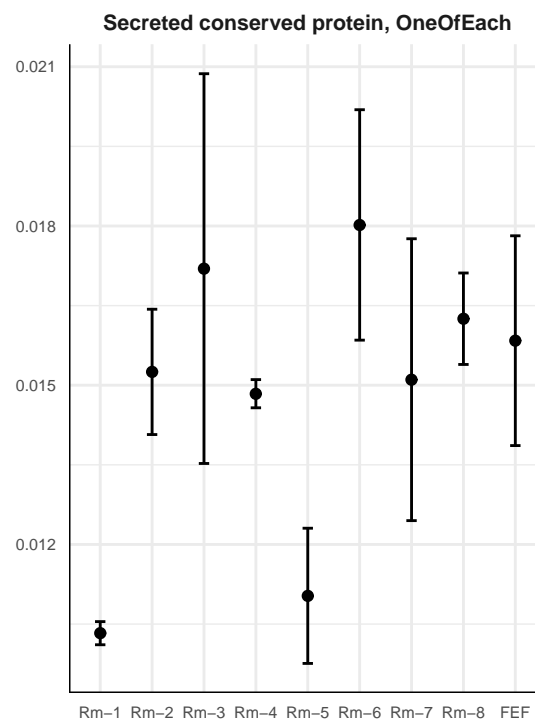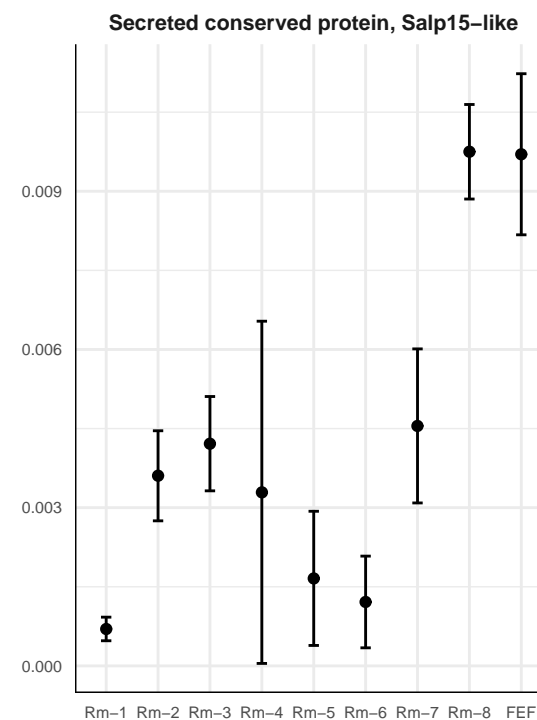

Supplement: Supplementary file 11 — Additional file 11: Fig. S8. Expression patterns of the secreted conserved proteins identified in the Rhipicephalus microplus saliva proteome throughout blood feeding. Each data point represents the average NSAF value for each protease family, with error bars denoting the standard error. [file 13071_2024_6136_MOESM11_ESM.pdf]
