# Supplementary material for: Changes in saliva protein profile throughout Rhipicephalus microplus blood feeding
Source: Parasit Vectors. 2024 Jan 27;17:36. doi: 10.1186/s13071-024-06136-5 (PMC10821567; doi:10.1186/s13071-024-06136-5)
Supplement: Supplementary file 9 — Additional file 9: Fig. S6. Amino acid alignment (ClustalW) of proteins identified within the TIL family of protease inhibitors identified in the Rhipicephalus microplus saliva proteome throughout blood feeding and the chymotrypsin inhibitor from Apis mellifera (PDB 1CCV). The putative P1 residues are highlighted by asterisks. The highly conserved residues are labeled in black, and the less conserved ones are in gray. [file 13071_2024_6136_MOESM9_ESM.pdf]

```
                                                            *
Rm-10457 : --------------------------------------------------
Rm-18594 : --------------------------------------------------
Rm-32449 : --------------------------------------------------
Rm-53166 : DTDGDSGVPVFKFPPDPADCGTNEVWKQCVSSTCAETTCTKRIVGPGCTY
Rm-53167 : DTDGDSGVPVFKFPPEAADCGSNEVWKQCVSSTCAETTCEKRFVGPSCTY
1CCV     : --------------------------------------------------

Rm-10457 : --------------------------------EGT--KCGEGEVYKENQS
Rm-18594 : --------------------------------QGTPQRCGSNETWKECVS
Rm-32449 : --------------------------------QRTPESCRPNETWKECVS
Rm-53166 : DCRYGCFCADGFYRNAEGNCVTVDQCPSADPDLGSSQQCGTNEEWKVCVS
Rm-53167 : DCIYGCYCADGFHRNAEGNCVSSDQCPPEEQNLGSSQQCGTNEEWKVCVS
1CCV     : ------------------------------------EECGPNEVFNTCG-
                              *
Rm-10457 : SSCGERKCGEPENGPRPCTLDLVSGCFCGDGLYRRSDNKCVSKRQCH---
Rm-18594 : SSCKEGTCEKPVVGP-ICTADCIYGCFCAEGFYRNQENLCVPLNECPGHH
Rm-32449 : SSCREGTCEKPVVGP-ACTADCIQGCFCAEGFYRNQEHLCVPLNECRGHH
Rm-53166 : SSCAETTCEKRTIGP-ACTADCRRGCYCSDGFHRNKEGACVTADQCPTV-
Rm-53167 : SSCAETTCEKRTIGP-ACTADCQRGCYCSDGFHRNKEGACVTADQCPTV-
1CCV     : SACAP-TCAQPKTRI--CTMQCRIGCQCQEGFLRNGEGACVLPENC----
```
