# Supplementary material for: Changes in saliva protein profile throughout Rhipicephalus microplus blood feeding
Source: Parasit Vectors. 2024 Jan 27;17:36. doi: 10.1186/s13071-024-06136-5 (PMC10821567; doi:10.1186/s13071-024-06136-5)
Supplement: Supplementary file 10 — Additional file 10: Fig. S7. Amino acid alignment (ClustalW) of proteins identified within the 8.9 kDa family of secreted conserved proteins identified in the Rhipicephalus microplus saliva proteome throughout blood feeding and the tick complement inhibitor CirpT1 from Rhipicephalus pulchellus (PDB 6RPT). The specific block of eight residues in the interaction interface between CirpT1 and C5 was identified [59] and residues are highlighted by asterisks. The highly conserved residues are labeled in black, and the less conserved ones are in gray. [file 13071_2024_6136_MOESM10_ESM.pdf]

```
Rm-149824 : NWNRVKYVNVRVNVTGRQCTYDNHSFTDKMNLNETCEERWCYPNKKTVIL :  50
Rm-18237  : -------------------------------------------------- :   -
Rm-26241  : ---------------------------------------------TSQER :   5
Rm-3247   : -------------------------------------------------- :   -
Rm-4943   : ------------------------------------------------QR :   2
6RPT      : --------------------------------------SAGLEVLFQGPM :  12

                     * *
Rm-149824 : LTCKDPKPGCHFKKGNKKFPKCCKTKCHKPRQPCVIKGTGDLLPDGQIRN : 100
Rm-18237  : ----TSG--ETFV----EVVDG----------KCVYQNI--TLEHHQSIQ :  28
Rm-26241  : QLQEDAKGECKAVRVTKKVPYG----------NCILNGT--TIKHGKTAD :  43
Rm-3247   : -DVQERG--HTYVTKNVTVEDG----------ACVYQRN--VIPDGETKA :  35
Rm-4943   : SKVRDPN--CAPKRYPDLLKPT----------NCTSNNK--TIRHGKTGD :  38
6RPT      : GDVQERG--HTYVTKNVTVEDG----------ACVYLRN--VIPNGETKA :  48

                                  *    * *        *  *
Rm-149824 : STD--PCVRYECHNG--NLTVTKC---DGADDKRCESSFANKTEPYPACC : 143
Rm-18237  : FES--PCQAWFCNADFHKIYFGGCT-PNPSLEN-C-VEVN-GNGTYPDCC :  72
Rm-26241  : SRSPYSCLGVYCWNG--TITPIRCKTPRPKDNDKFVHSRE-PG-DWPNCC :  89
Rm-3247   : LNN--PCILSTCYAAERKVNSTLC--PNIGVDEGCHVEWT-PDGVYPNCC :  80
Rm-4943   : TGS-AGCLGAYCWYG--HLITIPCPFGKPKNNEIYTYTRR-KNLTWPSCC :  84
6RPT      : LNN--PCVLSTCYAADRKVNSTLC--PNIGVDEGCHVEWT-PDGVYPNCC :  93

            *
Rm-149824 : GPATACVK-------------------- : 151
Rm-18237  : P-RLVCEGQSRG---------------- :  83
Rm-26241  : YWIRRCRNNTDPKRPMERSLRVSKIYHT : 117
Rm-3247   : P-KHVCPAAHATS--------------- :  92
Rm-4943   : YWTRRCTSEQE----------------- :  95
6RPT      : P-KHVCPSATASS--------------- : 105
```
