# Supplementary material for: Changes in saliva protein profile throughout Rhipicephalus microplus blood feeding
Source: Parasit Vectors. 2024 Jan 27;17:36. doi: 10.1186/s13071-024-06136-5 (PMC10821567; doi:10.1186/s13071-024-06136-5)
Supplement: Supplementary file 12 — Additional file 12: Fig. S9. Amino acid alignment (ClustalW) of CirpT1 homologs identified in the Rhipicephalus microplus saliva proteome and in the saliva proteome of other tick species and the tick complement inhibitor CirpT1 from Rhipicephalus pulchellus (PDB 6RPT). The specific block of eight residues in the interaction interface between CirpT1 and C5 was identified [59] and residues are highlighted by asterisks. The highly conserved residues are labeled in black, and the less conserved ones are in gray. [file 13071_2024_6136_MOESM12_ESM.pdf]

```
                                         *  *
Rm-3247     : ---------------------DVQERGHTYVTK-NVTVEDGACVYQRNVI : 28
Aam-175922  : --------------------GTLWHISDAYTGQAHVEVVDGKCVFQNRTL : 30
Aam-29382   : ---------------------DIQEHGHSYLTR-NVTVENGACIFERNTL : 28
AcajSigP-1  : ---------------------DIQEHGHSYLTR-NVTIENGACIFERNTL : 28
XP_0024020  : --------------------------EPPFS---DVVIVDGKCTYENHTV : 21
AAY66767.1  : --------------------------VPPYS---DVEIVDGKCKWGNYLV : 21
6RPT        : MKHHHHHHSAGLEVLFQGPMGDVQERGHTYVTK-NVTVEDGACVYLRNVI : 49

                                         *  *  *        *  *
Rm-3247     : PDGETKALNNPCILSTCYAAERKVNSTLCPNIGVDEGCHVEWTPDGVYPN : 78
Aam-175922  : GHHESIQLETPCQGWFCDIHAHTVSIGGCAPHALPDHCRTVNGS-GTYPN : 79
Aam-29382   : PDGETKALHDPCVIATCYAARREVNATLCRNFGVDPGCRFHWRNDGVYPQ : 78
AcajSigP-1  : PDGETKSLHDPCVIATCYAARREVNATLCRNFGVDPGCRVQWTPDGVYPQ : 78
XP_0024020  : ANGESLKWADPCQTWECNVEQRSFAVLGCGLVANPSGCELVRGT-GPYPD : 70
AAY66767.1  : PDGESLNLEDPCQTWQCSVEDRSFAVLGCGLIGNPYNCEIVRGT-GPYPN : 70
6RPT        : PNGETKALNNPCVLSTCYAADRKVNSTLCPNIGVDEGCHVEWTPDGVYPN : 99

                *
Rm-3247     : CCPKHVCPAAHATS------------- :  92
Aam-175922  : CCPQVVCDEQAQHNE------------ :  94
Aam-29382   : CCPTQVCDGTD---------------- :  89
AcajSigP-1  : CCPRQVCDRTD---------------- :  89
XP_0024020  : CCYRAVCP------------------- :  78
AAY66767.1  : CCYRTNCCGKPRYPRYWLGNDYRYPGK :  97
6RPT        : CCPKHVCPSATASS------------- : 113
```
